# The proprotein convertase *FURIN* is a novel aneurysm predisposition gene impairing TGF-β signalling

**DOI:** 10.1093/cvr/cvae078

**Published:** 2024-04-18

**Authors:** Zongsheng He, Arne S IJpma, Dianne Vreeken, Daphne Heijsman, Karen Rosier, Hence J M Verhagen, Jorg L de Bruin, Hennie T Brüggenwirth, Jolien W Roos-Hesselink, Jos A Bekkers, Danny F E Huylebroeck, Heleen M M van Beusekom, John W M Creemers, Danielle Majoor-Krakauer

**Affiliations:** Laboratory of Biochemical Neuroendocrinology, Department of Human Genetics, KU Leuven, Gasthuisberg O/N 06, Herestraat 49, Box 607, Leuven B-3000, Belgium; Department of Pathology, Erasmus MC University Medical Center, Dr. Molewater 40, PO BOX 2040, Rotterdam 3000 CA, The Netherlands; Department of Cardiology, Erasmus MC University Medical Center, Dr. Molewaterplein 40, PO BOX 2040, Rotterdam 3015 GD, The Netherlands; Department of Clinical Genetics, Erasmus MC University Medical Center, Dr Molewaterplein 40 PO BOX 2040, 3000CA Rotterdam, The Netherlands; Laboratory of Biochemical Neuroendocrinology, Department of Human Genetics, KU Leuven, Gasthuisberg O/N 06, Herestraat 49, Box 607, Leuven B-3000, Belgium; Department of Surgery, Erasmus MC University Medical Center, Dr. Molewaterplein 40, PO BOX 2040, 3000 CA Rotterdam, The Netherlands; Department of Surgery, Erasmus MC University Medical Center, Dr. Molewaterplein 40, PO BOX 2040, 3000 CA Rotterdam, The Netherlands; Department of Clinical Genetics, Erasmus MC University Medical Center, Dr Molewaterplein 40 PO BOX 2040, 3000CA Rotterdam, The Netherlands; Department of Cardiology, Erasmus MC University Medical Center, Dr. Molewaterplein 40, PO BOX 2040, Rotterdam 3015 GD, The Netherlands; Department of Cardiothoracic Surgery, Erasmus MC University Medical Center, Dr. Molewaterplein 40, PO BOX 2040, 3000 CA Rotterdam, The Netherlands; Department of Cell Biology, Erasmus MC University Medical Center, Dr. Molewaterplein 40, PO BOX 2040, 3000 CA Rotterdam, The Netherlands; Department of Cardiology, Erasmus MC University Medical Center, Dr. Molewaterplein 40, PO BOX 2040, Rotterdam 3015 GD, The Netherlands; Laboratory of Biochemical Neuroendocrinology, Department of Human Genetics, KU Leuven, Gasthuisberg O/N 06, Herestraat 49, Box 607, Leuven B-3000, Belgium; Department of Clinical Genetics, Erasmus MC University Medical Center, Dr Molewaterplein 40 PO BOX 2040, 3000CA Rotterdam, The Netherlands

**Keywords:** Aortic aneurysm gene, FURIN, TGF-β, Proprotein convertasePolygenic, genetic predisposition

## Abstract

**Aims:**

Aortic aneurysms (AA) frequently involve dysregulation of transforming growth factor β (TGF-β)-signalling in the aorta. Here, *FURIN* was tested as aneurysm predisposition gene given its role as proprotein convertase in pro-TGF-β maturation.

**Methods and results:**

Rare *FURIN* variants were detected by whole-exome sequencing of 781 unrelated aortic aneurysm patients and affected relatives. Thirteen rare heterozygous *FURIN* variants occurred in 3.7% (29) unrelated index AA patients, of which 72% had multiple aneurysms or a dissection. FURIN maturation and activity of these variants were decreased *in vitro*. Patient-derived fibroblasts showed decreased pro-TGF-β processing, phosphorylation of downstream effector SMAD2 and kinases ERK1/2, and steady-state mRNA levels of the TGF-β-responsive ACTA2 gene. In aortic tissue, collagen and fibrillin fibres were affected. One variant (R745Q), observed in 10 unrelated cases, affected TGF-β signalling variably, indicating effect modification by individual genetic backgrounds.

**Conclusion:**

*FURIN* is a novel, frequent genetic predisposition for abdominal-, thoracic-, and multiple aortic or middle sized artery aneurysms in older patients, by affecting intracellular TGF-β signalling, depending on individual genetic backgrounds.

## Graphical Abstract

**Graphical Abstract:**
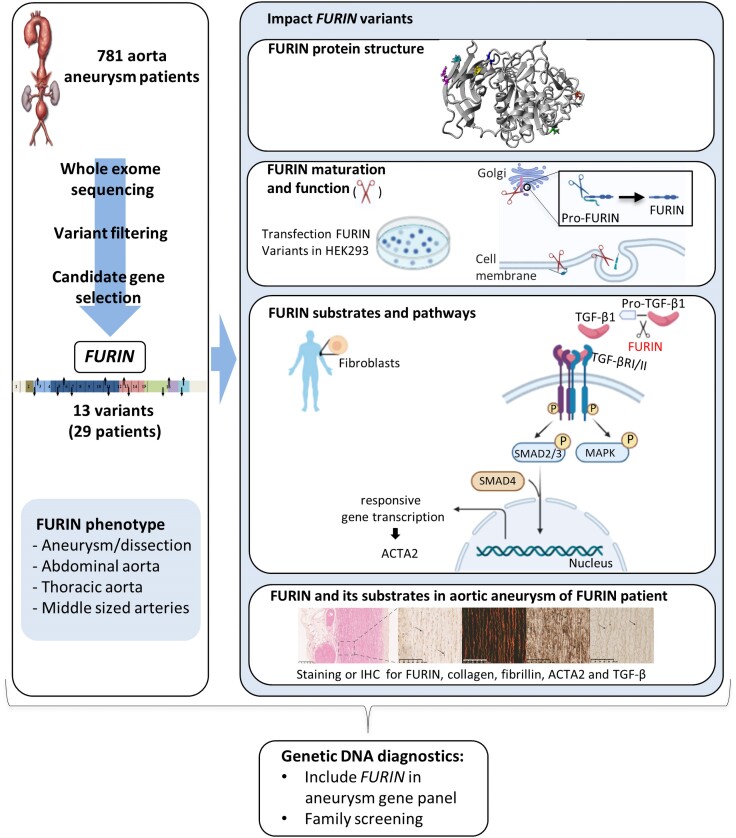

**Time of primary review: 42 days**

**See the editorial comment for this article ‘TGF-β signalling: the Dr Jekyll and Mr Hyde of the aortic aneurysms’, by S. Perrotta *et al.*, https://doi.org/10.1093/cvr/cvae245.**

## 1. Introduction

Aortic aneurysms (AA) are abnormal dilatations of the aorta, occurring more frequently in abdominal than in thoracic regions. AA represent important health care issues as they occur frequently in the population over 65 years and can result in life-threatening ruptures when undiagnosed and left untreated. Screening of high-risk populations was introduced to allow timely diagnosis and disease management while limiting mortality from rupture. Clinical guidelines recommend aneurysm screening for first-degree family members as their risk for AA is increased compared to the general population.^1,2^ While age, smoking, hypertension, and male gender are considered important risk factors, they are neither necessary nor sufficient, suggesting that interactions with genetic susceptibilities are important. Currently, in the majority of aneurysm cases, even familial aneurysms, no pathogenic variant in a known aneurysm gene is found. Only 2% of unselected AAA and 5% of unselected TAA patients have a (likely) pathogenic variant in one of the known AA genes. An explanation could be that most aneurysms have more complex genetics. One characteristic of this genetic complexity is modification of the pathogenic effects of genetic predispositions. Variability in age at onset and severity of clinical features between affected relatives in aneurysm families who share a pathogenic variant in a known aneurysm gene indicates that additional modifying genetic or non-genetic factors also have an impact on clinical penetrance. In this way, pathogenic variants in aneurysm genes contribute to complex aneurysm susceptibilities.^3–9^ In the majority of cases, similar to other common disorders, multiple genes—each with a small overall contribution and low relative risk—are expected to contribute to weakening of the aortic wall. A causative role for TGF-β-signalling loss-of-function mutations for aneurysms has been demonstrated in patients and several (conditional) knockout mouse models for e.g. *TGFBR1*, *TGFBR2*, *SMAD2, SMAD3*, and *SMAD4*.^10^ We selected *FURIN* as a candidate gene because it is expressed in the aorta, and is involved in TGF-β signalling.^11,12^ While the candidate gene approach has been used successfully to find novel susceptibility genes for other polygenic diseases such as obesity, it has not been used previously in aneurysm research.^13^ This approach is preferred over gene burden analyses, given that disease alleles also occur in unaffected individuals (non-penetrance) in the general population that would serve as the control group. Large population studies (e.g. UK biobank) recently showed that deleterious variants in genes for frequently occurring hereditary diseases, including aneurysm genes, are more frequent in the general population than expected.^14–17^

**Figure 1:**
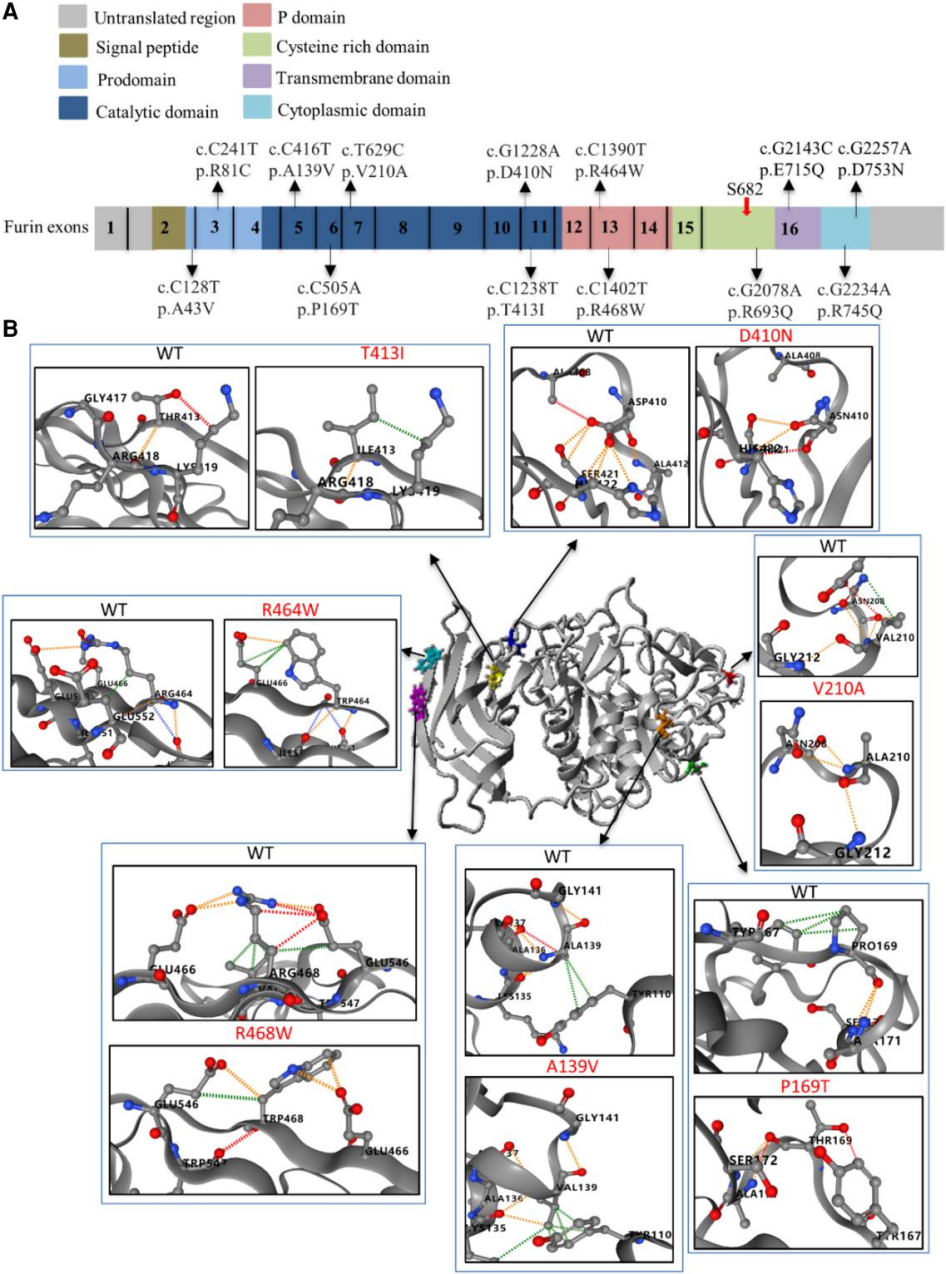
Crystal structure analysis of seven FURIN variants. (*A*) Variants in exons are indicated by thin arrows. The thick arrow indicates the shedding cleavage site (Ser 682). (*B*) Structures of catalytic and P domains of wild-type FURIN in grey. Red dotted line represents hydrogen bond; green dotted line represents hydrophobic interaction; yellow dotted line represents polar interaction.

FURIN is a subtilisin-like serine endoprotease synthesized as inactive proFURIN, translocated to the endoplasmic reticulum and upon trafficking along the secretory route becomes active in the *trans*-Golgi network (TGN) through autocatalytic cleavages.^11,18,19^ Active FURIN protein cycles between the TGN and the plasma membrane. At the cell surface a significant proportion of FURIN is shed as a soluble enzyme through a process that is not fully understood but involves cleavage of FURIN downstream the cysteine-rich domain preceding the transmembrane domain (*Figure 1A*). FURIN cleaves many different proprotein substrates, including proTGF-β, profibrillin and several procollagens, and the *FURIN* gene has been associated with several pathologies including hypertension and cardiovascular disease.^20,21^ For the identified *FURIN* variants, biological activity and impact on TGF-β signalling was tested.

## 2. Methods

### 2.1 Patients

The study population of this retrospective study consisted of 781 aneurysm patients consecutively diagnosed between January 2009 and July 2019 at the joint outpatient clinic of the Department of Clinical Genetics and Vascular Surgery at Erasmus MC.^22^ The study was approved by the Institutional Review Board (MEC 2012-078 and MEC-2013-265) of Erasmus MC. Appropriate written informed consent was obtained from all study participants and the investigations conformed to the principles outlined in the Declaration of Helsinki. AA patients were invited to evaluate genetic risk for relatives and receive recommendations for family screening by a clinical geneticist.^1,2^ This included ascertaining a detailed family history, physical examination, and DNA-sequencing with informed consent. Patients were classified as familial when an aneurysm was confirmed in at least one other family member (first or second degree) by medical records. When possible, DNA-testing of affected relatives was performed. Physical examination focused on features of syndromic aneurysms and connective tissue disorders, and included body build, hypermobility and skin signs. Skin fibroblasts were obtained from seven patients. Aortic tissue was available for family V7 (P169T FURIN variant) from open repair of an ascendens aneurysm (*Table 1* and Supplementary material online, *Table S1*).

**Table 1:** Predicted and observed effect of rare heterozygous FURIN variants in 29 unrelated aorta aneurysm patients

	*N*	Prediction		Frequency	FURIN activity and expression			Downstream effect in patient fibroblasts					
		Damaging effect^a^	CADD	MAF	activity	shed/intracellular	mature/(mature + pro)	F	T	P-	P-	A	L
								U	G	S	E	C	A
								R	F	M	R	T	B
								I	β	A	K	A	E
								N		D		2	L
c.128C > T, A43V	4	1/6	3.28	0.0026	↓↓	ns	ns	–	–	–	–		
c.241C > T, R81C	2	3/6	16.37	0.0016	↓↓	ns	ns	–	–	–	–	–	
c.416C > T, A139V	2	0/6	15.96	0.00036	↓↓	↓	↓	–	–	–	–	–	
c.505C > A, P169T	1	5/6	18.57	0.0006	↓↓	↓↓	↓↓	↓	↓↓	↓	**↓**	↓↓↓	V6
c.629T > C, V210A	1	3/6	15.10	0.000041	↓↓↓	↓↓	↓	↓↓↓	↓	↓↓	**↓**	↓↓↓	V7
c.1228G > A, D410N	1	3/6	21.3	–	↓↓↓	↓↓	↓↓	–	–	–	–	–	
c.1238C > T, T413I	2	2/6	13.84	0.00080	↓↓↓	↓↓↓	↓↓↓	–	–	–	–	–	
c.1390C > T, R464W	1	3/6	14.09	0.0013	ns	↓	ns	–	–	–	–	–	
c.1402C > T, R468W	1	3/6	17.00	0.000016	↓↓	↓↓	ns	–	–	–	–	–	
c.2078G > A, R693Q	1	0/6	0.3	–	↓↓↓	↓↓↓	↓↓	–	–	–	–	–	
c.2143G > C, E715Q	1	0/6	12.79	–	ns	ns	ns	–	–	–	–	–	
c.2234G > A,R745Q^b^	11	2/6	14.49	0.0012	ns	ns	ns	↓↓↓	↓↓↓	ns	ns	↓↓↓	V1
								↓	↓	↓	**↓**	↓↓↓	V2
								↓↓	↓	↓	**↓**	↓↓↓	V3
								↓	↓	↓↓	**↓**	↓↓↓	V4
								↓	↓	↓	**↓**	↓↓↓	V5
c.2257G > A, D753N	1	2/6	15.79	0.0004	ns	ns	ns	–	–	–	–	–	

^a^Damaging effect: number of programs predicting a damaging effect of the variant. Prediction programs used: Polyphen2, Align, SIFT, Mutation taster, Radial SVM, LR; CADD, combined annotation dependent depletion, a score that ranks genetic variants and is used to measure variant deleteriousness; MAF, minor allele frequency derived from ExAC NFE database.

^b^Downstream effects in fibroblasts of five unrelated patients (V1–V5). ↓ Decreased trends with significance compared to wild-type (*P* < 0.05); ↓↓, same, for *P* < 0.01; ↓↓↓, same, for *P* < 0.001; ↑↑↑: increased trends (*P* < 0.001); ns, not significant; –, not available.

### 2.2 Whole-exome sequencing data analysis

Whole-exome sequencing (WES) was performed using an Illumina HiSeq2500 or HiSeq4000 sequencer. Samples were enriched with either Agilent HaloPlex Target Enrichment System or the Agilent Technologies SureSelect Clinical Research Exome (CRE) capture kit. Reads were aligned to the reference genome (hg19) using BWA, and variant calling was conducted using the GATK software. Variants were annotated using Annovar.^23^ The average whole-exome coverage was >20, with 90% of the target regions covered >20 times. *In silico* prediction was performed using Polyphen2, Align, scale-invariant feature transform (SIFT), mutation taster, radial SVM, likelihood ratio (LR), and combined annotation dependent depletion (CADD) score. For variant identification only exonic missense, non-sense, stop-loss, frameshift and splice site variants with a MAF ≤ 0.01 in ExAC NFE were included. We compared the frequency of variants in *FURIN* (NM_002569) to the frequency of variants in known aneurysm genes in our study cohort. For that purpose, a diagnostic aneurysm gene panel that included the following genes was used*; ABL1, ACTA2, ADAMTS19, AEBP1, BGN, COL1A1, COL1A2, COL3A1, COL5A1, COL5A2, DCHS1, EFEMP2, ELN, FBN1, FBN2, FLNA, FOXE3, GATA5, HCN4, LMOD1, LOX, LTBP3, MAT2A, MFAP5, MYH11, MYLK, NOTCH1, NPR3, PLOD1, PRKG1, ROBO4, SKI, SLC2A10, SMAD2, SMAD3, SMAD4, SMAD6, TGFB2, TGFB3, TGFBR1, TGFBR2,* and *TLN1.*

A variant gene load of (likely) pathogenic variants (LPV) and of variants of unknown clinical significance (VUS) within *FURIN* was compared to these genes currently associated with aneurysms. These VUS and (L)PV variant gene loads were calculated by dividing the number of variants found in the gene by the coding sequence (CDS) length of the gene, and then multiplying this by 1000. Only exonic missense, non-sense, stop-loss, frameshift and splice site variants with a MAF ≤ 0.01 in ExAC NFE and, if available, a CADD score of 15 or higher were included.

### 2.3 FURIN protein structural modelling

A homology model was built using the X-ray structure of human FURIN (PDB file:4OMC, 100% identity) as a template,^24^ and an automatic YASARA script with standard parameters.^25^ Here, FURIN is a monomer, with amino acids 109–576 containing the previously mapped catalytic and P domain.^26^ Details of structural differences including predictions of changes in protein stability between wild-type and mutant FURINs are illustrated using the modelled structure by DynaMut^27^ (see Supplementary material online, *Table S2*).

### 2.4 Generation of human mutant FURIN constructs

Expression constructs for the production of human FURIN were pcDNA3 vector based (Invitrogen).^28^ The FURIN variants (R81C, P169T, V210A, D410N, T413I, R464W, R468W, E715Q, R745Q, and D753N, respectively) were generated by site-directed mutagenesis (Thermo Fisher Scientific; for the list of used primers, see Supplementary material online, *Table S3*). All constructs were tagged with a FLAG epitope, placed in-frame between the propeptide and catalytic domain, and each construct was verified by DNA sequencing (Applied Biosystems). All experiments were performed in triplicate.

### 2.5 Cell culture and transfections

Skin fibroblasts and HEK-293T were cultured in Dulbecco’s Modified Eagle’s Medium/Nutrient Mixture F-12 (Life Science) containing 10% foetal bovine serum (Perbio Science) and cultured at 37°C in humidified 5% CO_2_ atmosphere. HEK-293T were transfected using X-tremeGENE™ 9 DNA Transfection Reagent (Roche) according to the manufacturer’s protocol. Cells were grown in 6-well plates to 60–70% confluence before DNA transfection. Six microlitre X-tremeGENE Transfection Reagent was diluted in 100 μL of serum-free medium and incubated for 5 min at 24°C before adding it to 2 μg DNA. After incubation for 30 min at 24°C, this mix was added to the culture medium of the growing cells. After 24 h, transfected HEK-293T cells or medium were harvested for immunoblotting analysis or activity assay. Stimulation of skin fibroblasts with 1 ng/mL human TGF-β1 (R&D systems) was performed in serum-free medium for 8 h.

### 2.6 Lentiviral vector production and transduction in fibroblasts

Knockdown of *Furin* in human fibroblasts was achieved using lentiviral vectors. The vectors were produced in HEK293T cells by triple transfection with the transfer plasmid carrying a short hairpin *Furin* expression sequence (*shFurin*), the packaging plasmid pCMVΔR8.91, and the envelope plasmid encoding vesicular stomatitis virus G (VSV-G) protein (all Addgene). A GFP-transfer plasmid (Addgene) instead of the sh*Furin* plasmid was used as transfection control. The supernatant was harvested after 4 days, filtered (0.45 µm, VWR), concentrated (Millipore) and was used to transduce human cultured fibroblasts. Polybrene (5 µg/mL) was added to the concentrated viral particles and used to transduce fibroblasts. After 3 days incubation, the cells were selected with puromycin (1 µg/µL) for 5 days. RT-qPCR assay was performed to quantify the *Furin* mRNA levels in these cell lines. The extraction kit NucleoSpin® RNA (Macherey Nagel) was used to extract total RNA according to the manufacturer’s protocol. The iScript cDNA synthesis kit was used to generate the cDNA. RT-qPCR was carried out in triplicate using SYBR Green with a CFX Connect real time PCR system BIO-RAD. Samples were normalized to *Gapdh* expression, and data are represented as 2^−ΔΔCt^.

### 2.7 Immunoblotting

Transfected HEK-293T cells and patient fibroblast cells were lysed in cell lysis buffer (Cell Signaling Technology), supplemented with EDTA-free protease inhibitor cocktail and phosphatase inhibitors (Roche). After sonication, the preparation was centrifuged, and the supernatants were used as cell lysates. The protein concentration was measured using a Pierce™ BCA protein assays kit (Thermo Fisher Scientific). For TGF-β1 or secreted FURIN, conditioned medium without serum was collected after 24 h. One volume of 100% trichloroacetic acid (Sigma) was added to 4 volumes of conditioned medium. The proteins were precipitated overnight at 4°C and washed twice with −20°C acetone (Thermo Fisher Scientific). The samples were dried and resuspended in 1×SDS loading buffer prior to immunoblotting analysis. Proteins were resolved on 10% precast gels (Invitrogen) and electrophoretically transferred to a nitrocellulose membrane (Whatmann GmbH). After blocking with 5% non-fat milk in Tris-buffered saline with 0.2% Triton X-100, membranes were incubated with the primary antibody followed by the secondary antibody conjugated with horseradish peroxidase. After washing, the bands were visualized with enhanced chemiluminescence substrate (Perkin Elmer) and quantification of band intensities was performed using ImageJ. Membranes were re-probed with a blot restore solution following the manufacturer’s instructions (Thermo Fisher Scientific). Information about antibodies used is available (see Supplementary material online, *Table S4*).

### 2.8 RT-qPCR

For mRNA analysis, total RNA from cultured cells was isolated using NucleoSpin® RNA (Macherey-Nagel) according to the manufacturer’s protocol. Reverse-transcription of the RNA was performed using random primers iScript™ cDNA Synthesis Kit (Bio-Rad). Real-time PCR and data collection were performed with IQ SYBR Green supermix reagent (Bio-Rad) on a CFX connect instrument (Bio-Rad). Each sample was run in triplicate and normalized to the level of the transcript of the housekeeping gene *GAPDH*. Relative fold-changes were calculated using the ΔCT method with the equation 2ΔΔCT. The results are shown as x-fold changes compared to the control group average or relative levels to *GAPDH*. Primer sequences are available (see Supplementary material online, *Table S5*).

### 2.9 *In vitro* activity of FURIN variants

Soluble human FURIN was collected as a conditioned medium of transfected HEK-293T cells. The activity assay was performed in 96-well polypropylene microtiter plates in a final volume of 100 µL, containing 72.5 μL reaction buffer (100 mM HEPES, pH 7.0, 50 mM CaCl_2_, and 0.5% Triton X-100) and 25 µL conditioned medium. After a 30-minute preincubation at 37°C, 2.5 µL fluorogenic substrate (50 µM Pyr-RTKR-AMC) was added, reaction mixtures were incubated at 37°C, and fluorescence measurements (390 nm excitation, 460 nm emission) were taken under kinetic conditions every 10 min for 190 min in a Fluostar Galaxy fluorimeter. The fluorimetric output, which was obtained as relative fluorescence units (RFU), was plotted against time, and further processed. All fluorescence data are reported as the means of the measurements of three independent experiments, with error bar showing the standard deviations.

### 2.10 Histology and immunohistochemistry of aortic tissue

Ascending aortic aneurysm tissue of a patient with the *FURIN* P169T variant from family V16 was compared to paraffin-embedded healthy ascending aortic tissue from Erasmus MC Tissue Bank (Rotterdam, the Netherlands). Tissue sections (5 μm) were prepared for routine histology and immunohistochemistry (IHC). Sections were stained with haematoxylin and eosin (HE) as an overview stain, Miller’s Elastin (631075, VWR) as an elastin stain and Picrosirius red (PSR), interrogated by circular polarized light, as a collagen stain. IHC was performed for FURIN, fibrillin, TGF-β, ACTA2, SMAD4, and p-SMAD2 (see Supplementary material online, *Table S6*). For FURIN and TGF-β, following antigen retrieval and blocking for endogenous peroxidase [3% H_2_O_2_ (107209, Merck) in methanol (32213, Honeywell), 20 min] sections were incubated with 5% bovine serum albumin (BSA, A9647, Sigma) in PBS (pH 7.4, P4417, Sigma) to block non-specific antigen binding (1 h, RT). Sections were incubated overnight at 4°C with primary antibody diluted in 1% BSA. Antibody detection was visualized using the REAL Envision detection kit (K5007, DAKO), and sections were counterstained with haematoxylin. IHC for fibrillin, p-SMAD2, SMAD4, and ACTA2 was performed using the Ventana BenchMark Ultra automated staining system. For ACTA2 expression, quantification of DAB positive signal was performed using ImageJ. Images were first deconvoluted and thresholded for DAB positive areas. The average intensity of the thresholded areas was measured in the areas shown in *Figure 6P–R*, and five randomly selected areas for both patient and control. The control and patient sample slides each contained similar reference tissues that were used to normalize DAB intensities. The staining intensity was calculated by subtracting the corrected average intensity from the maximum intensity of 255 (0 = black and 255 = white).^29^ Statistics was performed using the Mann–Whitney *U* test.

### 2.11 Statistical analysis

Data are presented as mean ± SEM, unless otherwise indicated. A two-tailed unpaired Student’s *t*-test was conducted when comparing two groups. *P* < 0.05 was considered statistically significant. Statistics were calculated with Prism (GraphPad 7).

## 3. Results

### 3.1 *In silico* prediction of FURIN variants

*FURIN* was considered as a candidate gene because it is expressed in the aorta, and is involved in TGF-β signalling. Thirteen (heterozygous) missense variants in *FURIN* were observed in 29 (3.7%) unrelated patients of a large cohort of 781 consecutively diagnosed AA patients (*Table 1*). The strongest effects were predicted for variants R81C, A139V, P169T, V210A, D410N, R464W, and D753N, with a minor allele frequency (MAF) ≤ 0.001, combined with either a high CADD score (≥15) or predicted deleterious effect by three or more prediction programs. In 10 of 29 patients (34%), *FURIN* variants with the strongest effect were observed. Relatedness of the 11 cases with the R754Q variant was excluded by haplotype sharing analysis (data available upon request).

### 3.2 Structural and functional prediction of FURIN missense proteins

Nine *FURIN* variants (A43V, R81C, A139V, P169T, V210A, D410N, T413I, R464W, and R468W) were located within the structurally most conserved ectodomain: the pro-, catalytic, and P domains (*Figure 1A*). The prodomain acts as an intramolecular chaperone and assists in folding but also controls activity. The catalytic and P domains, are essential for endoproteolytic activity, the carboxyterminal domains play a role in intracellular trafficking and membrane anchoring.^19^

Modelling implications of amino acid substitutions using available crystal structures of catalytic and P domains showed no major effect on secondary structures (*Figure 1B*). However, some substitutions generated different local tertiary structure effects in these domains (*Figure 1B*). The P169T substitution predicts a reduction in steric hindrance leading to formation of new ionic interactions between Threonine and surrounding residues (*Figure 1B*). The additional hydroxyl in Threonine brings new hydrogen bonds into its side chain that is predicted to affect flexibility of the mutant protein. The P domain substitution R464W, as well as R468W substitution, adds new cyclic groups and largely increases local steric hindrance, thereby disturbing hydrogen bonding and ionic interactions with surrounding residues, probably leading to protein destabilization. These deductions are further supported by predicting the Gibbs free energy (ΔΔG) upon mutation, using machine-learning methods of DynaMut (see Supplementary material online, *Table S2*). Furthermore, catalytic domain variants V210A, D410N, and T413I also displayed altered local tertiary protein structures compared to wild-type FURIN structure.

Variants in the propeptide and carboxyterminal domains could not be analysed because they are not included in the crystal structure. The cysteine-rich domain, containing the R693Q variant, precedes the single-pass transmembrane domain and the cytoplasmatic domain (in which the E715Q, R745Q, and D753N variants are located), which are crucial for protein stability and intracellular trafficking. The variant R745Q is located in the sequence encoding the cytoplasmatic domain of FURIN. This domain is essential for TGN localization and endosomal trafficking to and from the plasma membrane.^30,31^ The replacement of the large, positively charged Arginine residue by the charge-neutral, polar Glutamine might disrupt electrostatic interactions with negatively charged amino acids. It might also affect functionality of nearby endocytosis motifs LI760 and YKGL765, and furthermore might impair binding to the cytoskeletal protein ABP-280 and hence cell surface tethering.^32^ Taken together, these variants were predicted to cause local tertiary structure changes which might affect protein folding, stability and/or catalytic activity of FURIN.

### 3.3 Impaired maturation, shedding, and proteolytic activity of FURIN variants

**Figure 2:**
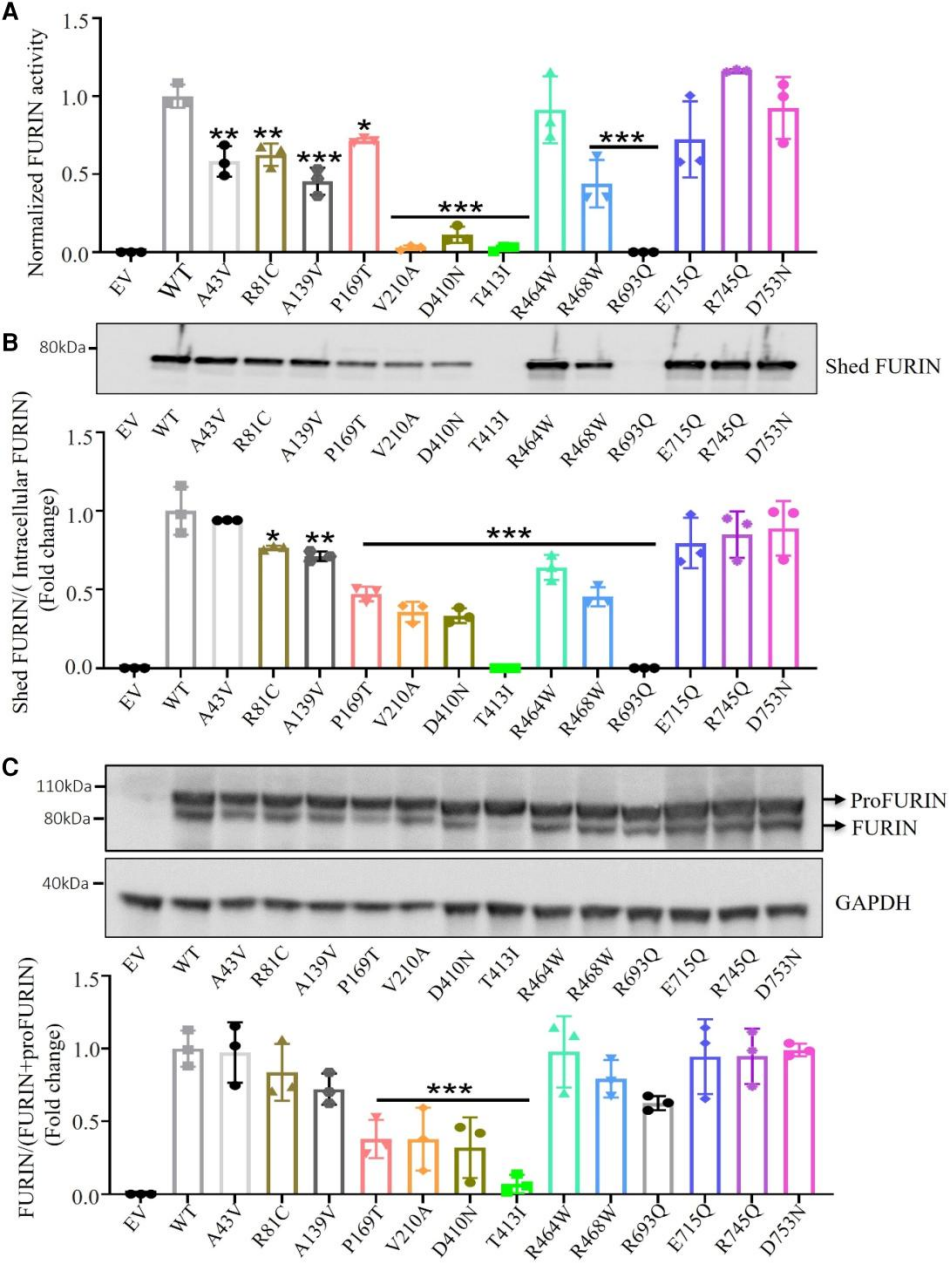
Furin maturation, shedding and activity in constructs of 13 *FURIN* variants. (*A*) The catalytic activity of shed FURIN expressed as the rate of change of relative fluorescence units. (*B*) Immunoblotting of shed FURIN in conditioned medium of transfected HEK293 T cells. Lower panel shows the quantification of shed FURIN/intracellular FURIN (proFURIN + FURIN) ratio. (*C*) Analysis of FURIN maturation in cell lysates of transfected HEK-293T cells. ProFURIN and mature FURIN are indicated. GAPDH was used as loading control. The lower panel shows the quantification of FURIN/(proFURIN + FURIN) ratio. EV, empty vector; WT, wild-type. All experiments were performed in biologically triplicate. Bars represent mean ± SEM. A one-way ANOVA followed by Dunnett’s *post hoc* test was performed. *P < 0.05, **P < 0.01, ***P < 0.001.

In 9 of the 13 FURIN variants an effect was observed on protein maturation within cells, shedding from cells, and/or proteolytic activity of shed FURIN in transiently transfected HEK293T cells. The processing of a fluorogenic substrate using conditioned medium from these cells (*Figure 2A*) showed more than 90% decrease in catalytic activity for variants V210A, D410N, T413I, and R693Q. A smaller, but significant reduction in activity was observed for A43V, R81C, A139V, P169T, and R468W.

Since activity was measured in conditioned medium that contains shed FURIN (84 kDa), we subsequently analysed the amount of shed FURIN by immunoblotting (*Figure 2B*).^33^ Six variants (P169T, V210A, D410N, T413I, R468W, and R693Q) showed strongly reduced amounts of shed FURIN, whereas shedding of A139V and R464W was moderately affected.

Determining ratios of inactive proFURIN (100 kDa) to cleaved FURIN (94 kDa) in cell lysates showed that A139V, P169T, V210A, D410N, T413I and R693Q variants had reduced amounts of processed FURIN (*Figure 2C*).

### 3.4 Impaired intracellular TGF-β signalling in fibroblasts of patients with FURIN variants

**Figure 3:**
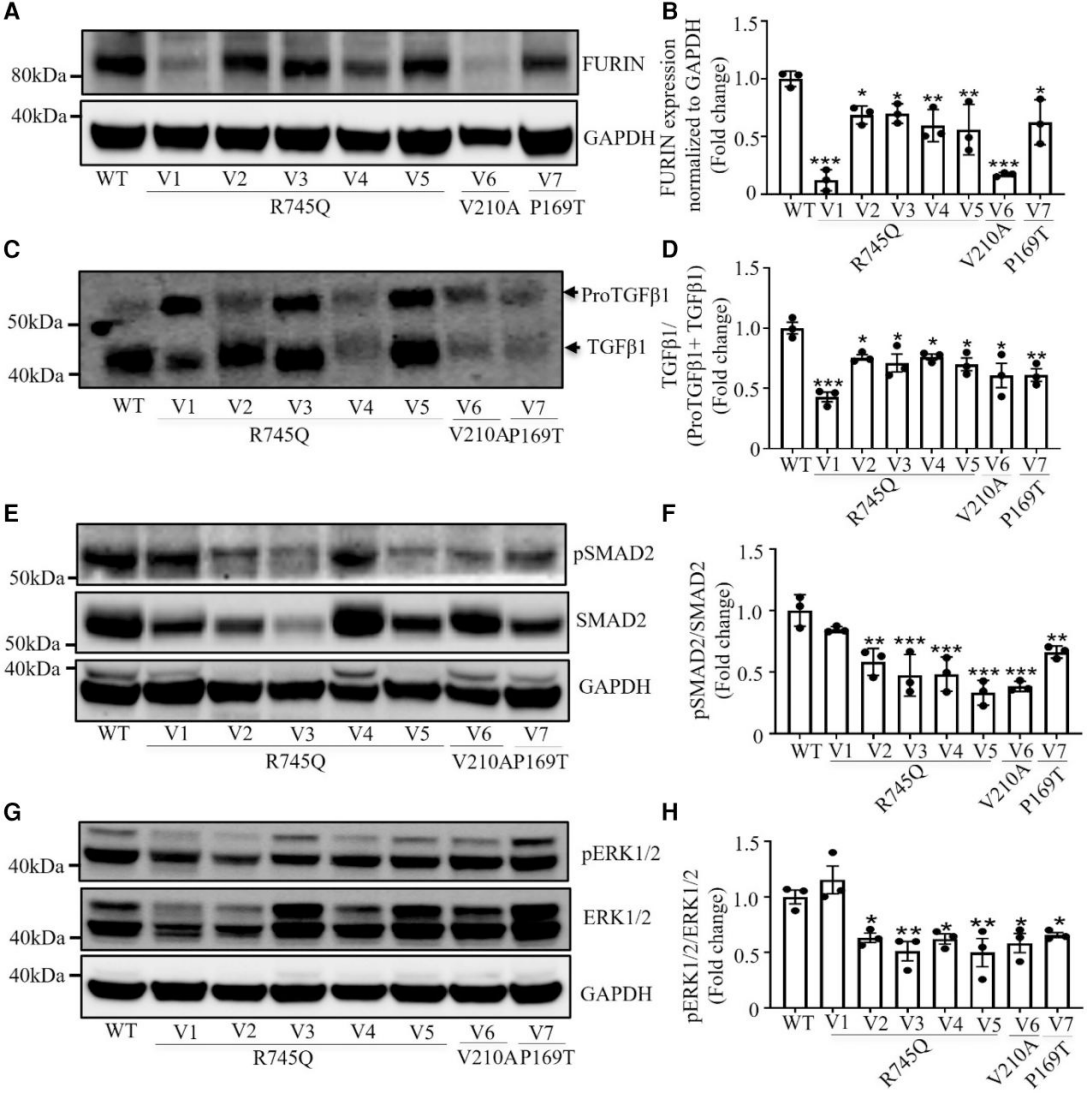
TGF-β maturation and signalling in fibroblasts of seven patients with heterozygous *FURIN* variants. Immunoblotting and quantification in skin fibroblast of: (*A, B*) FURIN. (*C, D*) TGF-β maturation. (*E, F*) phosphorylation of SMAD2. (*G, H*) ERK1/2 phosphorylation. GAPDH was used as a control. All experiments were performed in biologically triplicate. Bars represent mean ± SEM. WT, wild type; V, variant. A one-way ANOVA followed by Dunnett’s *post hoc* test was performed. *P < 0.05, **P < 0.01, ***P < 0.001.

**Figure 4:**
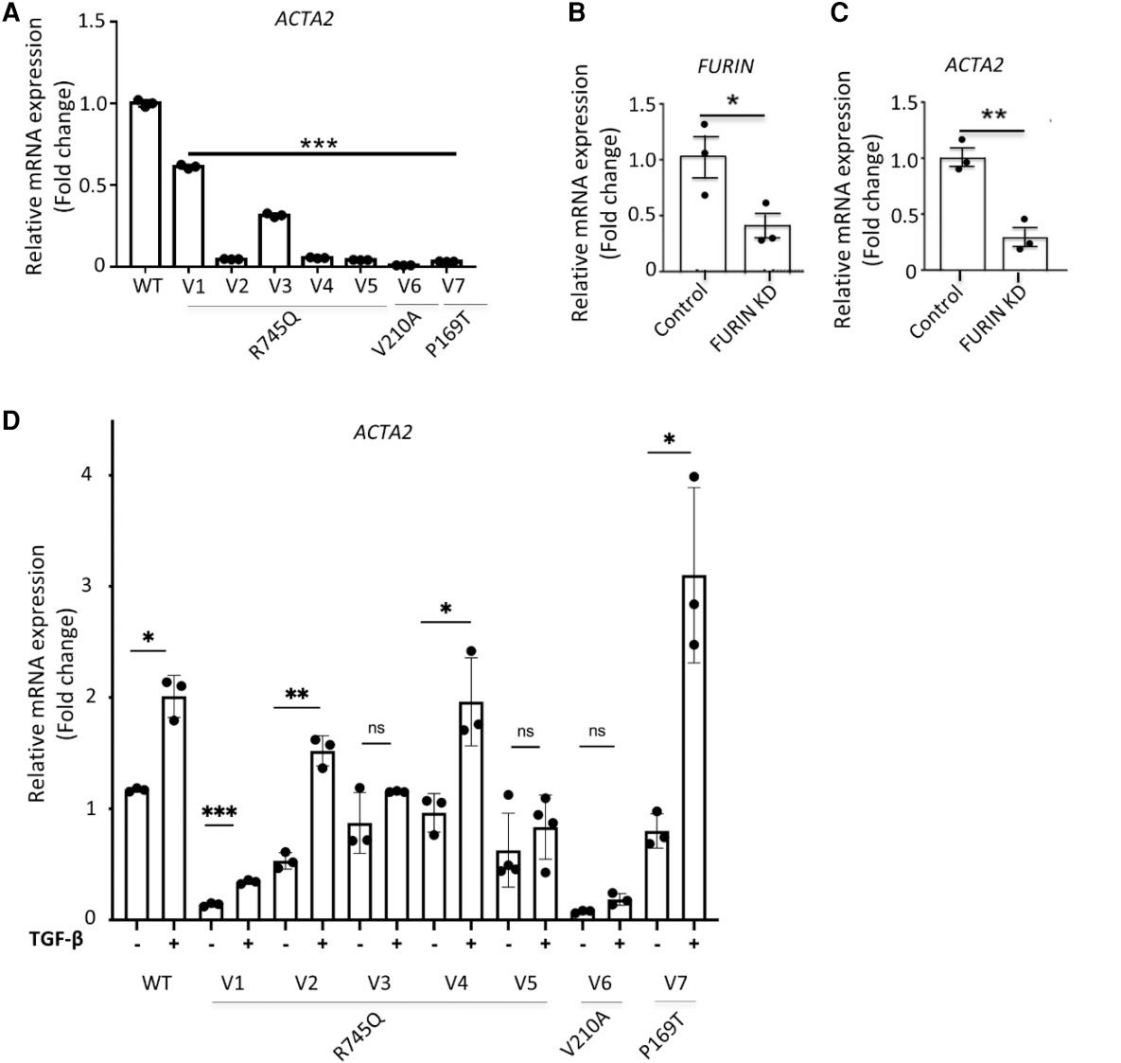
Relative mRNA expression of ACTA2 in fibroblasts of seven patients with heterozygous *FURIN* variants and after knock-down of FURIN. Relative mRNA expression of ACTA2 (*A*), Relative mRNA expression of FURIN and of ACTA2 in FURIN knock down cells with GAPDH as a control (*B*), Relative mRNA expression of ACTA2 with or without stimulation with exogenously added 1 ng/mL TGF-β1 for 8 h (*C*). WT, wild-type; V, variant; *ACTA2*, smooth muscle actin alpha 2. All experiments were performed in biologically triplicate. Bars represent mean ± SEM. A one-way ANOVA followed by Dunnett’s *post hoc* test was performed for (*A*). A two-tailed Student’s *t*-test was performed for (*B–D*). *P < 0.05, **P < 0.01, ***P < 0.001.

Analysis of the FURIN variants in the previous sections indicated that specific amino acid changes had functional consequences. However, the unphysiologically high levels in transiently transfected HEK293T and the heterologous cellular context may preclude other and more subtle downstream effects from being unveiled. Therefore, we used patient-derived skin fibroblasts to analyse the effect on TGF-β maturation and its signalling. Skin fibroblasts from seven heterozygous *FURIN* aneurysm patients were available for this study; from the patients with the P169T and the V210A variant, and from five unrelated patients with the R745Q variant (*Figures 3* and *4*). Steady-state levels of FURIN were reduced to a variable extent in all examined patient fibroblasts (*Figure 3A* and *B*).

Processing of proTGF-β1 to mature TGF-β1 was impaired in fibroblasts of all patients (*Figure 3C* and *D*). Decreased phosphorylation of downstream SMAD2 and ERK1/2 (a protein kinase component of non-SMAD signalling) was observed in five out of the six patients (*Figure 3E–H*). A significant decrease in mRNA expression of the TGF-β-regulated gene *ACTA2* was observed in all (*Figure 4A*). To confirm that impaired FURIN function directly leads to reduced TGF-β signalling and target gene expression, FURIN was knocked down in control fibroblasts (*Figure 4B*). The ∼60% reduction in FURIN resulted in ∼75% reduction in *ACTA2* expression (*Figure 4C*). Despite the reduced TGF-β signalling, patient cell lines V1, V2, V4, and V7 remained responsive to exogenously added TGF-β (*Figure 4D*). V3, V5, and V6 also showed an increase in *ACTA2* expression after stimulation, albeit not significantly. Together, these data show that the cell lines have impaired but not blocked TGF-β signalling.

### 3.5 Furin, collagen, fibrillin, TGF-β, and ACTA2 in the ascending aorta of a patient with the *FURIN* variant P169T

**Figure 5:**
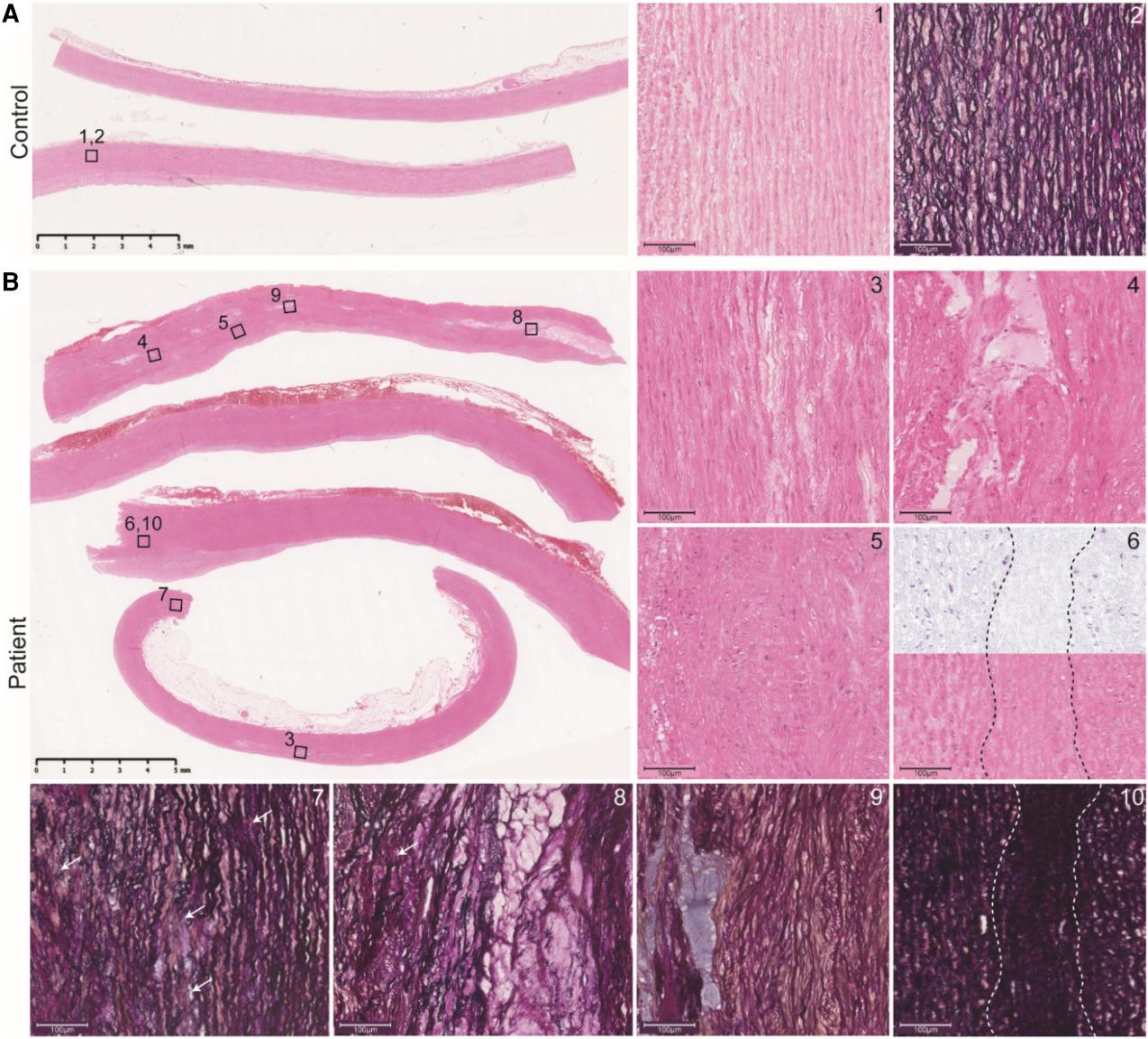
Medial degeneration in ascending aorta of a patient with the P169T variant. Ascending aorta sections of a control (*A*) and patient (*B*) stained with haematoxylin–eosin (HE) or Millers Elastin. (*A*) Overview of the control aorta. Detailed areas: (1, 2) organization of smooth muscle cells and elastin layers. (*B*) Overview of the patient aorta. Detailed areas: (3, 4) intra- and translamellar MEMA, (5) smooth muscle cell disorganization, (6) band like loss of smooth muscle cells (between dotted lines) in deconvoluted haematoxylin staining (upper part) and HE (lower part), (7–9) different degrees of elastin fragmentation (arrows) and/or loss (10) lamellar medial collapse in the same area as depicted in (6).

The affected aorta of the patient showed moderate to severe characteristics of an aortic aneurysm, including multifocal medial mucoid extracellular matrix (MEMA) with intra- and translamellar expansions. Other observed signs of aortic aneurysm pathology were extensive elastin fragmentation/loss, and smooth muscle cell disorganization/loss, resulting in lamellar medial collapse (*Figure 5B*, panels 3–10).^34^

**Figure 6:**
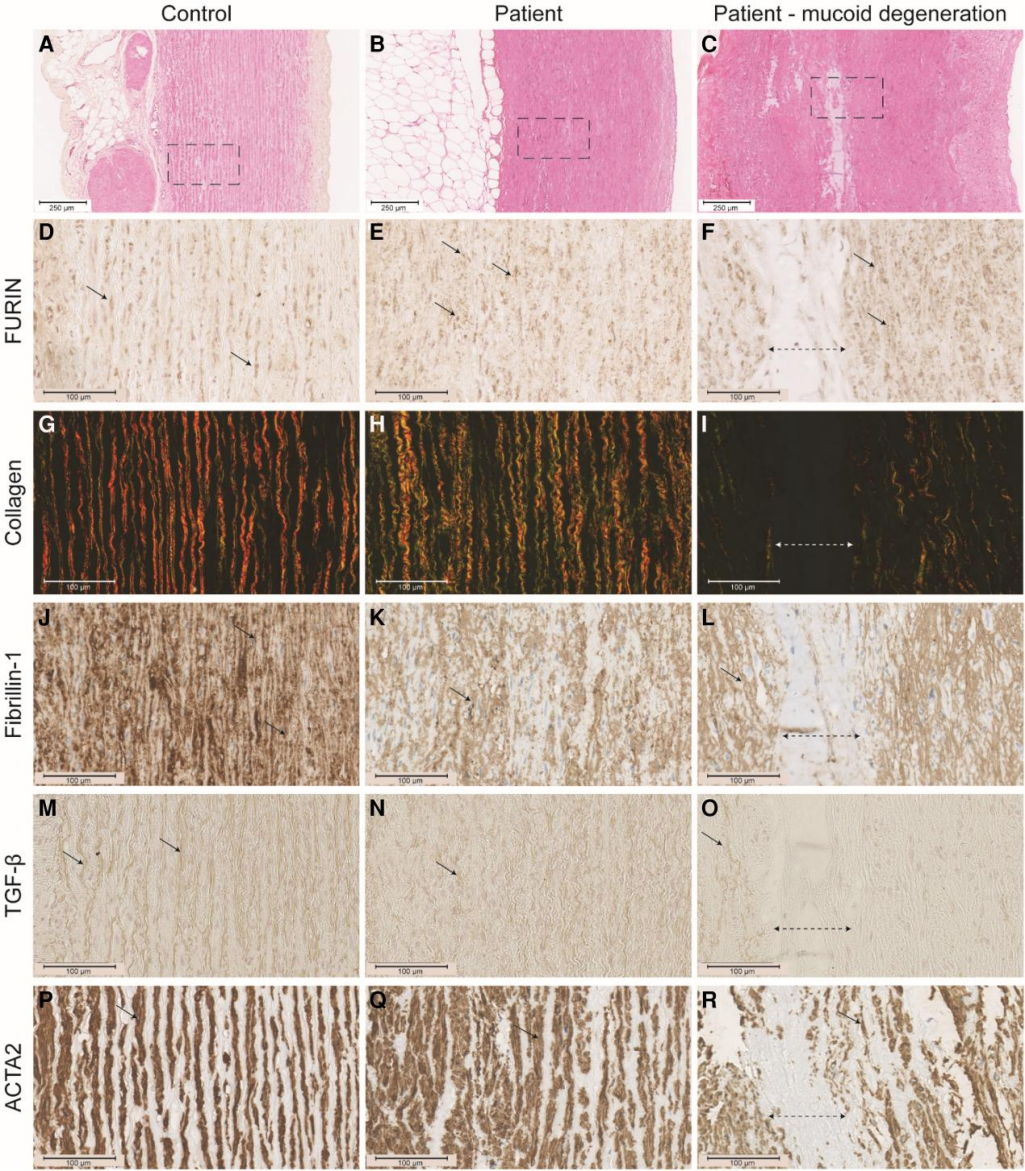
Furin, collagen, fibrillin-1, TGF-β, and ACTA2 in the ascending aorta aneurysm of a patient with the P169T *FURIN* variant. Overview (HE) of control tissue (*A*), patient tissue without (*B*) and with (*C*) mucoid degeneration. Selected detailed areas stained for FURIN (*D–F*), collagen (*G–I*), Fibrillin-1 (*J–L*) *E*) TGF-β (*M–O*) and ACTA2 (*P*, *R*). Arrows indicate positive staining. Dotted line with arrow heads indicates mucoid degeneration.

The patient showed FURIN in intracellular vesicle-like structures while the control showed perinuclear FURIN consistent with trans-Golgi network localization (*Figure 6D–F*). Patient tissue showed more thin (green) collagen fibres than thick (red) collagen fibres upon interrogation of Picrosirius red (PSR) with circular polarized light (*Figure 6G–I*). Fibrillin signals were decreased (*Figure 6J–L*). A moderate decrease of TGF-β was observed (*Figure 6M–O*). ACTA2 intensity was decreased in patient aorta (staining intensity: 138 ± 5.27 (patient) vs. 162 ± 1.38 (control), *P* = 0001) (*Figure 6P–R*). FURIN, collagen, fibrillin, TGF-β, or ACTA2 were absent from areas with mucoid degeneration (*Figure 6F, I, L, O, R*).

### 3.6 Clinical characteristics of 29 aneurysm patients with *FURIN* variants

In 21 of 29 (72%) aneurysm patients with a *FURIN* variant allele, multiple aneurysms occurred. Twelve (41%) patients had an aneurysm or dissection of the thoracic aorta, and 8 (28%) had both AAA and a dilation of the thoracic aorta. Midsize arteries were affected in 17 (58%) of 29 patients, and ruptures or dissections occurred in nine (28%) patients (*Table 2* and Supplementary material online, *Table S1*). The spectrum of vascular phenotype in the 29 index patients included 21 juxta-/infrarenal aneurysms, 4 descendens and 9 ascendens or arch aneurysms, while iliac aneurysms were observed in 9 patients. Two patients had a type-B dissection, femoral or popliteal artery aneurysms. Splenic or lienal artery aneurysms, and bilateral carotid aneurysms were all observed once. Nine patients (31%) had an aneurysm in other locations than the abdominal (juxta or infrarenal) aorta.

**Table 2:** Aorta aneurysms in 29 aneurysm index cases with a FURIN variant

	Aortic aneurysm patients with a *FURIN* variant		
	Total *n* = 29	Male *n* = 21	Female *n* = 8
Abdominal aorta aneurysm	22	19	3
Rupture/dissection	5/3	4/2	1/1
Thoracic aorta dilatation	12	8	4
Middle-sized artery aneurysm/dilatation	17^a^	13	4
Multiple aneurysms	21	16	5
Abdominal aneurysm mean age at diagnosis y (range)	63 (32–82)	63 (32–82)	58 (49–67)
Familial aneurysms	16 (55%)	12 (57%)	4 (44%)

^a^8/17 midsized aneurysm patients had ≥2 midsized artery dilations.

A range of connective tissue features (including those observed in Marfan and Loeys-Dietz syndrome) like a Marfanoid habitus (arm span to body length ratio >1.04), scoliosis or kyphosis, pectus excavatum, and/or joint hypermobility with an elevated Beighton score were observed in 12 (41%) patients. Eight patients (26%) had a velvety skin and/or skin translucency, hyper-extensibility or dystrophic scarring. Of the eleven unrelated patients with the *FURIN* R745Q variant, six reported familial aneurysms. Most R754Q variant patients (10/11) had an infrarenal aneurysm and eight had multiple aneurysms. Extravascular features of the R745Q patients included three with skin features, five had skeletal signs (scoliosis, kyphosis, pectus excavatum) and three hypermobility (luxation, Beighton score >6/9).

DNA-testing of the known aneurysm genes in a clinical setting showed that one patient (V16) had a likely pathogenic variant (LPV) of *TGFΒR2* as well as the *R745Q* variant in *FURIN*. The female patient was diagnosed at age of 81, with a large fast-growing AAA, and also an aneurysm of the ascending of 43 mm and a dilatation of 33 mm of the descending. She had a brother who died at age 76 years during an open repair of an abdominal aneurysm. *FURIN* segregation analysis could not be performed because exome data were not available. One of her three middle aged children with the *TGFΒR2* LPV, had unilateral fusiform dilation of a renal artery at age 56. No characteristic extravascular features of Loeys–Dietz syndrome were observed in the index and offspring with the *TGFΒR2* LPV.

### 3.7 Prevalence estimates

To underline the contribution of *FURIN* variants to AA, the prevalence of *FURIN* variants was compared to the prevalence in the study population of variants selected in the same way as the *FURIN* variants, in the 42 aneurysm genes currently used in the diagnostic aorta aneurysm panel. In this way the adjusted variant load for *FURIN* and for the 42 aneurysm genes was calculated in the study population genes. This showed that, when corrected for coding sequence (CDS) length, *FURIN* had a variant load comparable to the *TGFBR2, DSCH1*, *NOTCH1*, and *COL1A2* genes in the study population (*Table 3*).

**Table 3:** Variant gene load for FURIN and selected aneurysm-related genes

Gene	Variant gene load	Transcript	Coding sequence length
TGFB2	21.07	NM_001135599	1329
GATA5	18.43	NM_080473	1194
SMAD6	12.74	NM_005585	1491
NPR3	8.61	NM_001204375	1626
MYH11	5.56	NM_001040114	5940
FBN2	5.49	NM_001999	8739
PLOD1	4.58	NM_000302	2184
COL3A1	4.54	NM_000090	4401
COL5A1	4.53	NM_001278074	5517
COL1A1	4.10	NM_000088	4395
MYLK	4.00	NM_053025	5745
TGFBR2	3.93	NM_001024847	1779
LMOD1	3.88	NM_012134	1803
DCHS1	3.84	NM_003737	9897
NOTCH1	3.78	NM_017617	7668
**FURIN**	3.77	NM_002569	2385
COL1A2	3.17	NM_000089	4101
AEBP1	3.16	NM_001129	3477
FBN1	3.13	NM_000138	8616
FOXE3	3.13	NM_012186	960
COL5A2	3.11	NM_000393	4500
SKI	2.74	NM_003036	2187
LTBP3	2.56	NM_001130144	3912
TGFB3	2.42	NM_003239	1239

### 3.8 Family histories of aneurysms

Familial aneurysms (FAA) occurred in more patients with a *FURIN* variant than would be expected (FAA in 16 (55%) patients with a *FURIN* variant, compared to the expected ∼20% of FAA in abdominal—or thoracic aneurysm cases), endorsing that *FURIN* variants may increase familial susceptibility to aneurysms (*Table 2* and Supplementary material online, *Table S1*). An aneurysm was confirmed in the medical records of 22 affected relatives, showing that 17 relatives had an abdominal and 5 had a thoracic aneurysm or dissection (16 were deceased). Family screening for aneurysm of 34 relatives above age 55 years from 14 families (12 FAA, 2 SAA) showed that one of the 18 relatives examined by abdominal echography had an abdominal aneurysm (the 77-year-old father of V10 suffering from heart failure and lung fibrosis). No DNA was available. None of the relatives who had a CT scan of the total aorta, had an aneurysm (an overview of family screening is presented in Supplementary material online, *Table S1*). In two families cerebral aneurysms were reported.

### 3.9 Segregation of *FURIN* variant in three families

Since few affected relatives were alive and consented for DNA testing, familial segregation of FURIN variants could only be established in three examined families.

**Figure 7:**
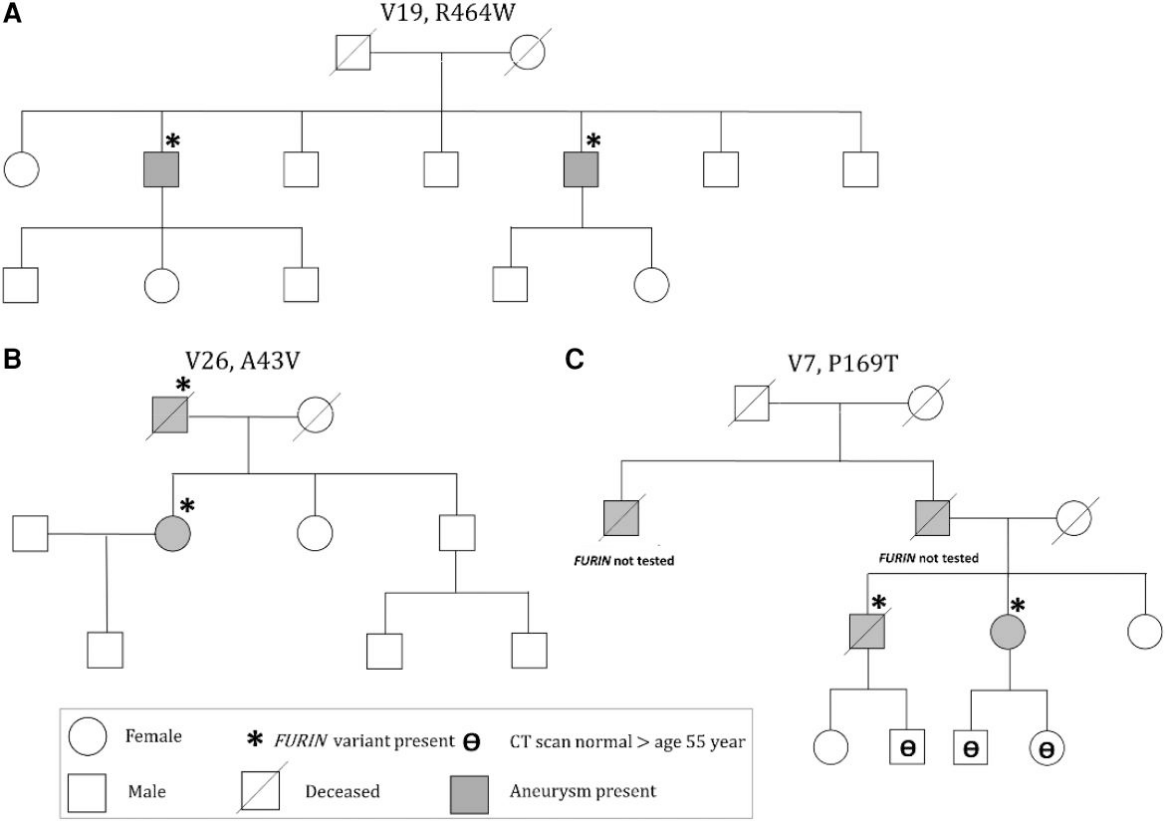
Three families with segregation of aneurysms and FURIN variants.

The *FURIN* variant P169T was identified in a 71-year old female (V7) with an aneurysm (40 mm) of the aorta ascendens after her brother had a replacement of the aorta ascendens at age 68 (51 mm). She had a translucent skin, signs of atrophic scarring, and was treated for hypertension and hypercholesterolaemia. The deceased father and one of his brothers were reported to have had an AAA around age 65–70 (*Figure 7A*).

V19, the patient and his brother both had an abdominal aneurysm and the R464W variant. The index case had an open repair of a suprarenal aneurysm at age 66 (*Figure 7B*). Surveillance for renal failure showed an infrarenal aneurysm of 34 mm in his brother at age 65. The index case had a scoliosis, arachnodactyly, and an inguinal hernia.

In V26, both the female patient with a type-B dissection and a TEVAR at age 56, and her father with an abdominal aneurysm had the A43 V variant (*Figure 7C*). The index case had translucent skin, varices, a herniated disk, and suffered from jaw dislocations and sleep apnoea. Her father had an open repair at age 74 for a juxtarenal and infrarenal saccular aneurysm. He had herniation of an abdominal scar. In two other families, no segregation of FURIN variants could be established; a cousin with a dilation of the ascending aorta in family V4 tested negative (see Supplementary material online, *Figure S1A*). In family V11, the tested maternal uncle (with a AAA, TAA and popliteal aneurysm) did not have the R693Q variant (see Supplementary material online, *Figure S1B*).

## 4. Discussion

*FURIN* was identified as a novel late onset genetic predisposition gene for aneurysm. This gene was selected as a candidate gene for AA based on the biological effect of FURIN on pro-TGF-β maturation and subsequent TGF-β signalling, a key signalling pathway associated with aortic aneurysm. Some of the FURIN variants had stronger predicted effects than others and all variants were tested in our functional assays. Our results demonstrated that heterozygous *FURIN* variants can impair proTGF-β processing and TGF-β maturation, and hence compromise TGF-β signalling. Effects on important features of aneurysm formation were observed: TGF-β signalling, collagen and fibrillin dynamics, and smooth muscle functioning. Furthermore, compromised non-canonical intracellular signalling was observed through the MAPK/ERK pathway. One of the actions of the non-canonical pathway is actinomyosin (ACTA2) regulation and subsequent regulation of smooth muscle cell functioning in the aorta. Corroborating results showed a decrease in ACTA2 in patients’ fibroblasts and cell constructs. In addition, aneurysm tissue showed an altered collagen structure and decreased levels of fibrillin and ACTA2. Fibrillin functions as a scaffold for elastin, is an important regulator for bioavailability of active TGF-β, and mutations in fibrillin are a well-known cause for aneurysms in Marfan syndrome. The above results are in line with a recent Smad-4 knock out mouse model study which showed increased aneurysm formation as a consequence of decreased Smad4 activity, and the decrease in TGF-β signalling in Loeys–Dietz syndrome.^35–37^ In contrast, other studies into the effect of Fibrillin mutations pointed into the direction of an increase of SMAD3 signalling suggesting that the stage of the aneurysm, the specific genetic defect or differences between human and mice determine to some extend whether SMAD and ERK are either activated or not.^36,38,39^ The altered intracellular localization of FURIN in vesicle-like structures in the examined aneurysm tissue of the patient with the P169T variant, might result from disturbed intracellular FURIN vesicle trafficking from ER to Golgi since auto activation is a prerequisite for exit out of the ER.^18^ The structure prediction models showed that the P169T variant could affect the flexibility of the mutant protein. This could explain the impaired maturation of proFURIN into FURIN, a process that takes place in the ER.

*FURIN* variants affected TGF-β signalling depending on the individual genetic background of the patients. These findings expand the spectrum of aneurysm genes affecting TGF-β signalling*. FURIN* variants were identified in 3.7% of this study population with abdominal-, thoracic aorta, and middle sized arteries aneurysms, similar to the vascular features in vascular Ehlers–Danlos, Marfan, and Loeys–Dietz syndrome. Extravascular musculoskeletal and skin signs were observed, as in other genetic aneurysms with modified TGF-β signalling, albeit less prominent than in Marfan and Loeys–Dietz syndromes, where the extravascular signs are often the distinguishing features.

### 4.1 Polygenic effects

The hallmark of common complex disorders is that they are the result of the interplay of (multiple) genetic factors and other risk factors increasing overall susceptibility for the disorder.^40^ While many of the genetic and non-genetic risks are abundantly present in the general population, disease only occurs in individuals with a specific combination of these factors. Likewise in Mendelian disorders, interplay of risk factors eventually determine phenotype, non-penetrance, difference in age at onset, and delineate the range of severity of clinical features.

In families with aortic aneurysms where a pathogenic variant in a known aneurysm gene has been identified, the individual genetic background plays an important role. As characteristic for these familial aneurysms are the variability in the location of the aortic dilatation or dissection in affected relatives.^3–7^

The polygenic nature of FURIN-related aneurysms was best illustrated with the highly variable *in vivo* effect of the recurrent R745Q variant in unrelated cases and the difference between *in vitro* (identical genetic background) and *in vivo* (unique patient genetic background) experiments. The R745Q variant did not show an effect in the *in vitro* assays, whereas the fibroblasts of five unrelated patients showed significantly impaired TGF-β maturation and downstream signalling, indicating that the genetic background of patients contributed to the observed effects. In one of the five R745Q patients, less pronounced effects were observed reflecting interaction(s) with individual genetic backgrounds. Further studies into interactions between genetic factors are needed.

### 4.2 Prediction of the impact of *FURIN* variants

We observed that none of the applied pathogenicity-prediction methods completely reflected the biological effects. For some variants like P169T and V210A the *in silico* predictions were consistent with the observed effects in the experiments, other variants such as A43V and R693Q had a strong reduced FURIN protease activity in the *in vitro* assays, while based on prediction programs (CADD of respectively 3.28 and 0.3) these variants would not be expected to have any effects. Based on these observations we conclude that the best approach to assess the effect of any variant may be to combine pathogenicity predictions with activity assay testing.

The current contribution of other aneurysm genes to aorta aneurysm is based on the occurrence only of (L)PV, excluding patients with variants of unknown clinical significance (VUS). Performing functional testing on a larger scale as we have carried out for *FURIN*, may show that a genetic predisposition can be identified in a larger part of aneurysms patients. Accordingly, in our study population, the VUS and (L)PV variant load of *FURIN* was similar to that of *NOTCH1, COL1A2*, and *FBN1* in the study population.

Currently, the contribution of aneurysm genes to aorta aneurysm is probably an underestimate, because it is based only on the occurrence of (L)PV, excluding patients with variants of unknown clinical significance (VUS). Functional testing of all VUS in these genes, as we have done for *FURIN*, may show that a genetic cause can be identified in a larger part of the AAA patients. Finding genes that predispose to aneurysms is important because we can more accurately identify which relatives carry the genetic susceptibility and have a high risk of developing aneurysms and will benefit most from screening, early detection, and treatment of the weakening of the aorta.

### 4.3 Clinical implications

*FURIN* was identified as a frequent, late-onset genetic aneurysm risk factor. Therefore, we propose to add *FURIN* to the diagnostic gene panel for aneurysms. Adding analysis of *FURIN* to the diagnostic gene panel for aneurysm, will identify a larger group of aneurysm patients and families who may develop a complex vascular phenotype based on TGF-β dysregulation, and these patients may need corresponding clinical management. Patients with heterozygous rare *FURIN* variants need clinical surveillance for aneurysms and dissections of thoracic and abdominal aorta and mid-sized arteries. Therefore, recommendations for family screening of *FURIN* patients should include whole-aorta screening, similar to genetic thoracic aneurysm and dissection (TAAD) syndromes. *FURIN* variant alleles were observed in both familial and apparently sporadic cases, clearly indicating that one cannot rely on family history alone to identify the genetic AA that carry an elevated risk for relatives.

We observed modulation of effects by individual genetic background, which may lead to asymptomatic aneurysm in relatives. This may explain in part underreporting of familial disease, along with unawareness of the health status of relatives, misdiagnosis in relatives and small family size.

Further studies are needed to validate these results in other large study populations, explore the role of additional genetic influences and other risk factors on the extent of the aortic, other vascular or extravascular lesions, and to explore if *FURIN* variant affect the outcome of aneurysm repair and survival. Eventually the goal is to improve diagnosis and treatment of aneurysm patients and this can be achieved through designing polygenic risk algorithms for AA, incorporating known risk factors like age, gender, and smoking with information on variants in aneurysm predisposition genes.

### 4.4 Limitations

The study population size did not allow identification of the factors contributing to the polygenic effect of *FURIN* variants on AA, such as variants in other, aneurysm or TGF-β pathway genes, or genes that have not yet been associated with aneurysms. Similarly, investigating effects of additional known risk factors for aneurysms were out of reach for this study. Segregation within families of *FURIN* variants was hampered by the late onset of disease, with most affected relatives no longer alive. The number of patient derived fibroblasts and aneurysm tissue available for experiments was limited as it is difficult to obtain this type of material from patients. Patient derived skin fibroblasts were used in this study for *in vivo* experiments to interrogate the effects of *FURIN* variants. Preferably vascular smooth muscle cells would have been interrogated, but while it was attenable to obtain patient derived skin fibroblasts, obtaining vascular SMCs from patients was not possible. There may be an underreporting of VUS in other aneurysm genes, because some DNA diagnostics performed in the past may not have included all genes that are currently tested.

## 5. Conclusions

This is the first report that *FURIN* is a genetic predisposition for aneurysms. The effects of *FURIN* variants include abdominal and thoracic aortic and mid-sized artery aneurysms and multiple aneurysms at older age. In familial and (apparent) sporadic aneurysms *FURIN* effects may be modulated by individual genetic backgrounds. Adding analysis of *FURIN* to the diagnostic gene panel for aneurysm, will identify a larger group of patients and families who may have a TGF-β regulated complex vascular phenotype and need corresponding clinical management.

**Translational perspective**

Dysregulation of TGF-β signalling is associated with aortic aneurysms. The proprotein convertase FURIN, which functions include TGF-β maturation, was identified as a novel genetic aneurysm predisposition and was shown to be modulated by the individuals genetic background. Adding *FURIN* to the diagnostic gene panel for aneurysms, will improve DNA diagnostics, prediction, identification, and clinical management of complex genetic vascular phenotypes.

## Supplementary Material

cvae078_Supplementary_Data

## Data Availability

The datasets supporting the current study have not been deposited in a public repository but are available from the corresponding author on request.
