# Supplementary material for: The proprotein convertase FURIN is a novel aneurysm predisposition gene impairing TGF-β signalling
Source: Cardiovasc Res. 2024 Apr 18;120(17):2278–92. doi: 10.1093/cvr/cvae078 (PMC11687399; doi:10.1093/cvr/cvae078)
Supplement: cvae078_Supplementary_Data [file cvae078_supplementary_data.docx]

**Supplementary data accompanying He *et al*:**

**The proprotein convertase *FURIN* is a novel aneurysm predisposition gene impairing TGF-β signaling**

**Short title: The novel TGF-β aneurysm gene *FURIN***

**Supplement Figure 1. Two families with no segregation of aneurysms and *FURIN* variants.**


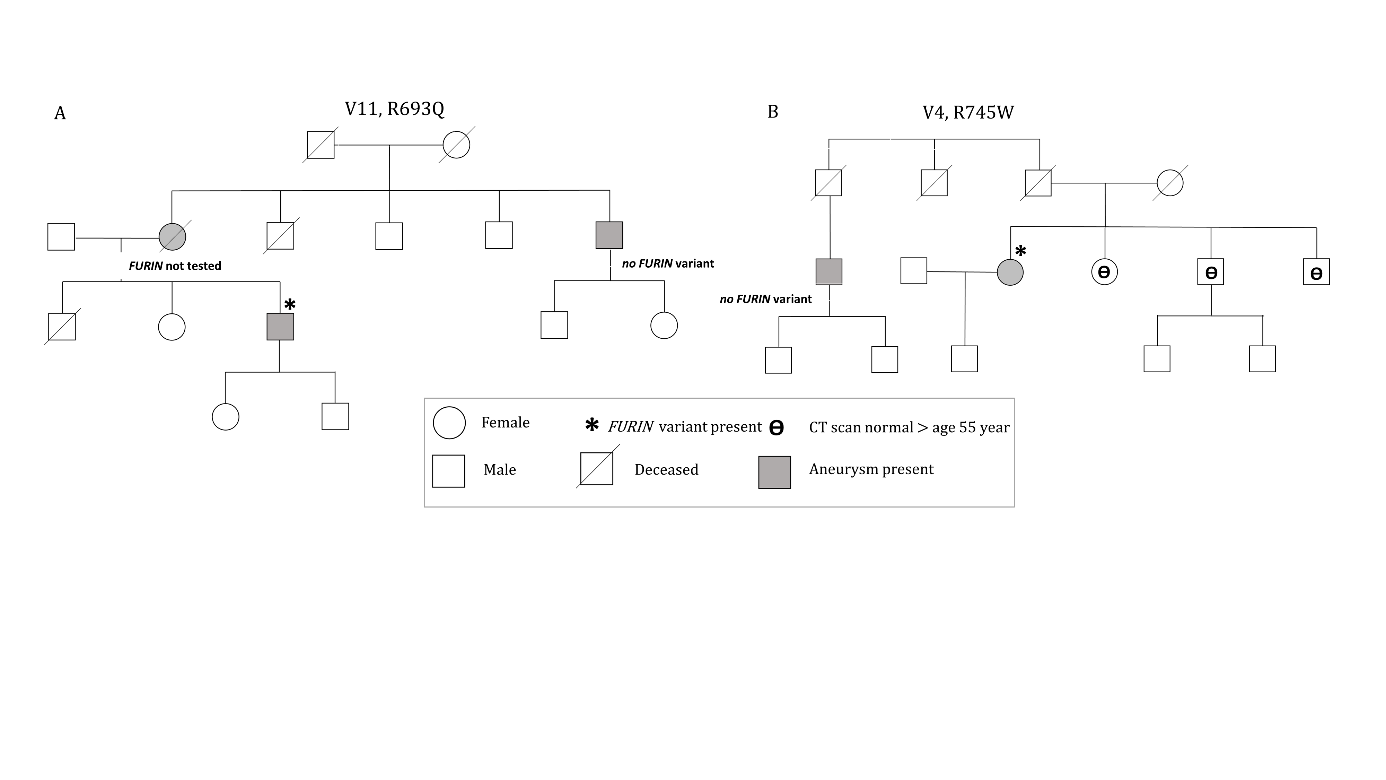


**Supplement raw blots for Figure 2B and 2C**

**
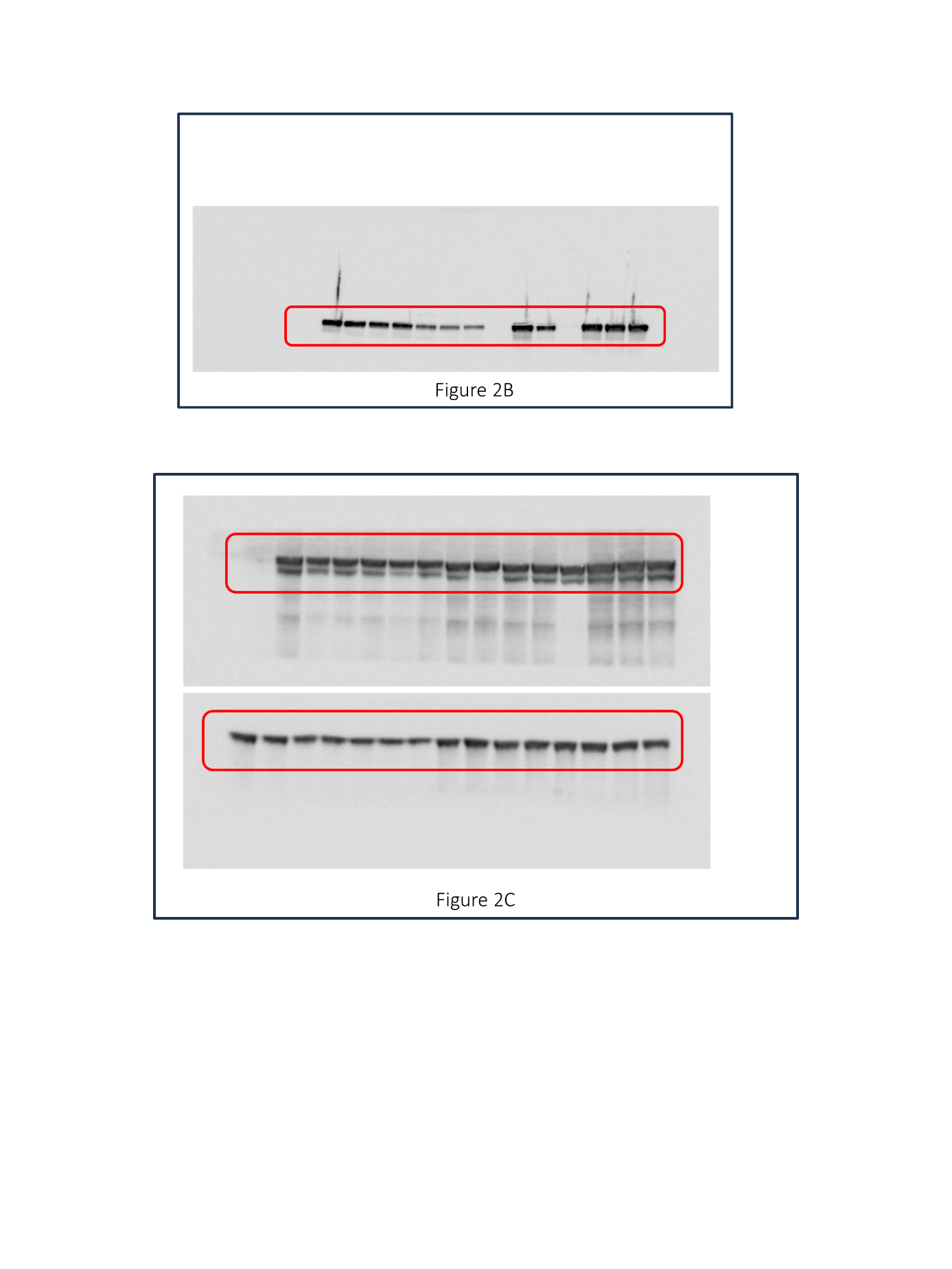
**

**Supplement raw blots for Figure 3A and 3C
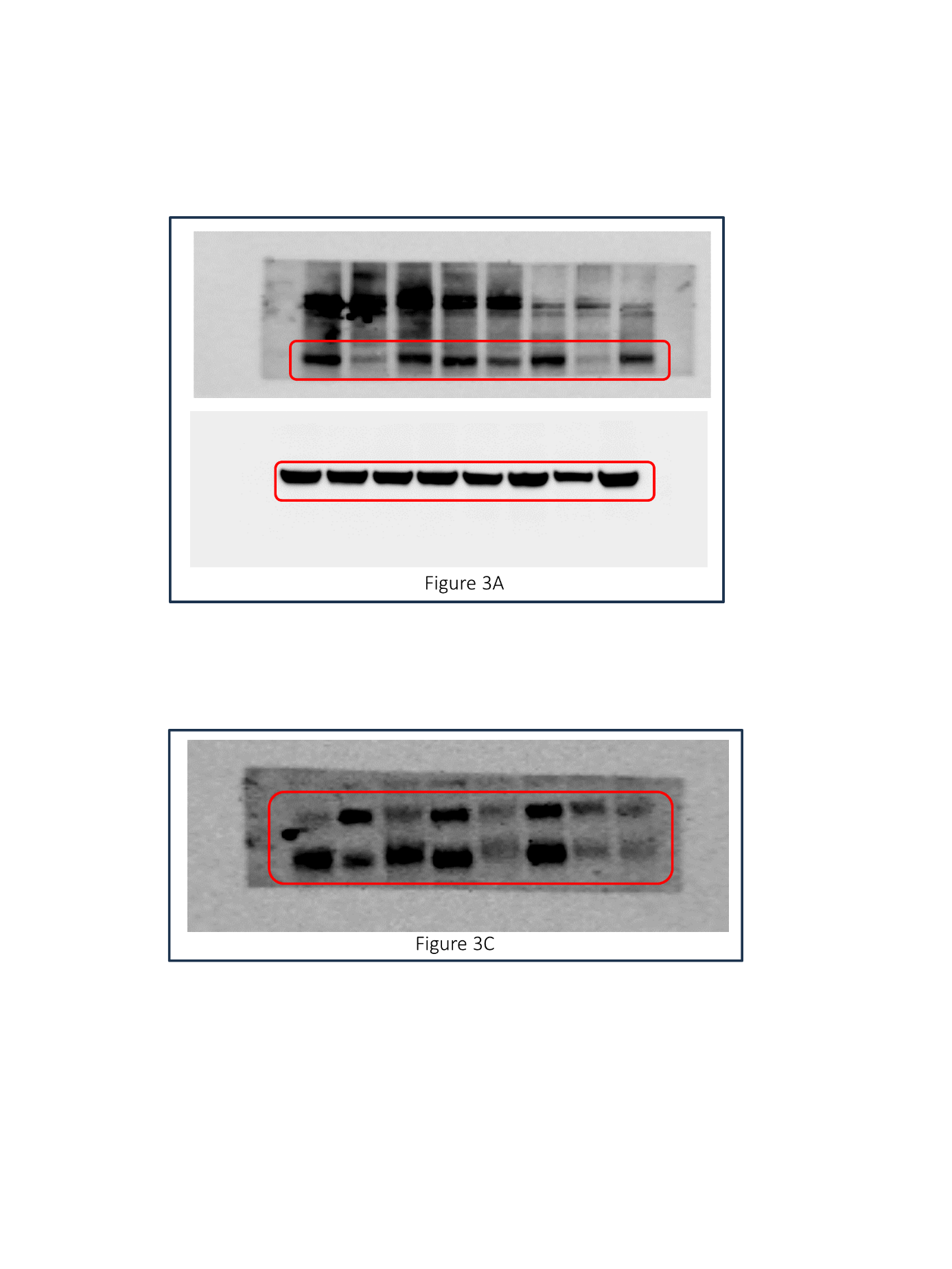
**

**Supplemental raw blots for Figure 3E and 3G**

**
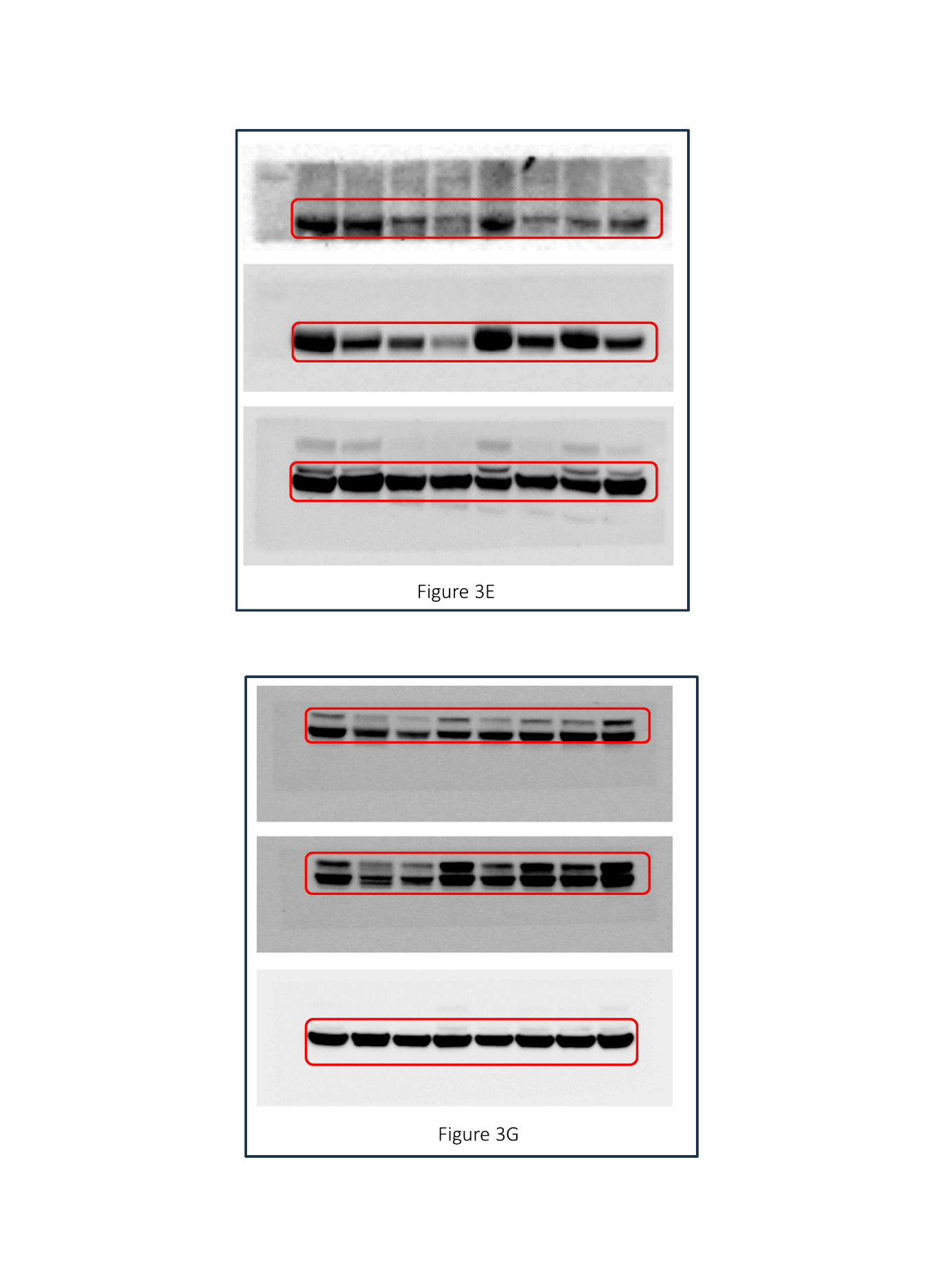
**

**Supplement Table S1**

Due to table size, supplement table 1 is added at the end of the supplementary data file.

| *FURIN* variant | Predicted ΔΔG (kcal/mol) | Prediction |
| --- | --- | --- |
| A139V | -0.62 | Destabilizing |
| P169T | -0.39 | Destabilizing |
| V210A | -1.62 | Destabilizing |
| D410N | -0.24 | Destabilizing |
| T413I | -0.57 | Destabilizing |
| R464W | -0.42 | Destabilizing |
| R468W | -0.01 | Destabilizing |

**Supplement Table S2. Predictions of changes in protein stability based on sequence and structure in different FURIN variant proteins.**

**Supplemental Table S3. Forward and reverse primers used for PCR-based detection of point mutations of *FURIN.***

| Primers | Sequence | Length (nt) |
| --- | --- | --- |
| a-R81C-Forward | TCCCTGTCGCCTCACTGCCCGCGGCACAGCC | 31 |
| a-R81C-Reverse | GGCTGTGCCGCGGGCAGTGAGGCGACAGGGA | 31 |
| a-P169T-Forward | CAAAACTGGCCCCAGTATCATAATTGCCTGC | 31 |
| a-P169T-Reverse | GCAGGCAATTATGATACTGGGGCCAGTTTTG | 31 |
| a-V210A-Forward | GGCCAACAACGGTGCCTGTGGTGTAGGTG | 29 |
| a-V210A-Reverse | CACCTACACCACAGGCACCGTTGTTGGCC | 29 |
| a-D410N-Forward | CACCTCAATGCCAACAACTGGGCCACCAATG | 31 |
| a-D410N-Reverse | CATTGGTGGCCCAGTTGTTGGCATTGAGGTG | 31 |
| a-T413I-Forward | CCAACGACTGGGCCATCAATGGTGTGGGCCG | 31 |
| a-T413I-Reverse | CGGCCCACACCATTGATGGCCCAGTCGTTGG | 31 |
| a-R464W-Forward | AAAGACATCGGGAAATGGCTCGAGGTGCGGA | 31 |
| a-R464W-Reverse | TCCGCACCTCGAGCCATTTCCCGATGTCTTT | 31 |
| a-R468W-Forward | CGGCTCGAGGTGTGGAAGACCGTGACC | 27 |
| a-R468W-Reverse | GGTCACGGTCTTCCACACCTCGAGCCG | 27 |
| a-E715Q-Forward | CCCTCACACCTGCCTCAGGTGGTGGCCGGCC | 31 |
| a-E715Q-Reverse | GGCCGGCCACCACCTGAGGCAGGTGTGAGGG | 31 |
| a-R745Q -Forward | CTGGCTTTAGTTTTCAGGGGGTGAAGGTGTAC | 32 |
| a-R745Q -Reverse | GTACACCTTCACCCCCTGAAAACTAAAGCCAG | 32 |
| a-D753N-Forward | AAGGTGTACACCATGAACCGTGGCCTCATCT | 31 |
| a-D753N-Reverse | AGATGAGGCCACGGTTCATGGTGTACACCTT | 31 |

**Supplement Table S4. Antibodies used for immunoblotting.**

| Antibodies | Source | Identifier |
| --- | --- | --- |
| Rabbit monoclonal anti-phospho-Smad2 (Ser465/467)  Rabbit monoclonal anti-Smad2  Rabbit monoclonal anti-phospho-ERK1/2 (Thr202/Tyr204)  Rabbit monoclonal anti-ERK1/2  Rabbit monoclonal anti-GAPDH  Goat polyclonal anti-human LAP TGF­β1  Mouse monoclonal anti-Furin (MON-152)  Swine anti-rabbit horseradish peroxidase (HRP)-conjugated 2ary antibody  Rabbit anti-goat HRP-conjugated 2ary antibody  Rabbit anti-mouse HRP-conjugated 2ary antibody | CST  CST  CST  CST  CST  R&D system  Enzo Life  DAKO    DAKO  DAKO | Cat# 3108  Cat# 5339  Cat# 4377  Cat# 4695  Cat# 2118  Cat# AF-246-SP  ALX-803-017-R100  Cat# P0217  Cat# P0449  Cat# P0260 |

**Supplement Table S5. Forward and reverse primers used for quantitative RT-PCR.**

| Primers | Sequence (5´-3´) | Length (nt) |
| --- | --- | --- |
| FURIN-Forward | AGGATGAATCCCAGGTGCT | 19 |
| FURIN-Reverse | CTCGGGAGGGTGAAGAGTG | 19 |
| ACTA2-Forward | CAGCCAAGCACTGTCAGGAAT | 21 |
| ACTA2-Reverse | CCATCACCCCCTGATGTCTG | 20 |
| GAPDH-Forward | CCCATCACCATCTTCCAGGA | 20 |
| GAPDH-Reverse | CTTCTCCATGGTGGTGAAGAC | 21 |

**Supplement Table S6. Primary antibodies immunohistochemistry.**

| **Antigen** | **Reference** | **Manufacturer** | **Titer** | **Antigen retrieval** |
| --- | --- | --- | --- | --- |
| Furin* | MON-152 | Enzo life | 1:200 | 20 min, 97 ^o^C in citrate (pH 6.0) |
| Fibrillin-1 | MAB2502 | Merck Millipore | 1:100 | 32 min, 97 ^o^C in Cell conditioning solution 1 (CC1, Ventana) |
| TGFβ* | MO-C400009A | Anogen | 1:5000 | 20 min, 97 ^o^C in citrate (pH 6.0) |
| p-SMAD2 | 3108 | Cell Signaling Technologies | 1:100 | 32 min, 97 ^o^C in Cell conditioning solution 1 (CC1, Ventana) |
| SMAD4 | SC-7966 | Santacruz | 1:100 | 32 min, 97 ^o^C in Cell conditioning solution 1 (CC1, Ventana) |
| ACTA2 | AB5694 | Abcam | 1:2500 | 32 min, 97 ^o^C in Cell conditioning solution 1 (CC1, Ventana) |

*****Furin was manually stained, all other by the Ventana BenchMark Ultra

**Supplement Table S1. Clinical features and family histories of 31 unrelated abdominal aorta aneurysm patients with a *FURIN* variant.**

| **ID** | ***FURIN variant*** | **Sex** | **Aneurysm** | **Repair** | **Complication** | **Phenotype** | **Familial (FAA)** | **Multiple aneurysms** | **N relatives screened** | **Affected relatives** | **Familial segregation *FURIN variant*** |
| --- | --- | --- | --- | --- | --- | --- | --- | --- | --- | --- | --- |
|  |  |  |  |  |  |  | **Sporadic (SAA)** |  |  |  |  |
| **V1** | R745Q | M | Infrarenal | EVAR |  | emphysema | SAA | no | none | none | not tested |
|  |  |  |  | FEVAR |  |  |  |  |  |  |  |
| **V2** | R745Q | F | Femoral | Coiling |  | varices | Familial cerebral aneurysm (CAA) | yes | none | Multiple 2^nd^  degree CAA | not tested |
|  |  |  | Splenic |  |  | velvety skin |  |  |  |  |  |
|  |  |  |  |  |  | scoliosis |  |  |  |  |  |
|  |  |  |  |  |  | luxation |  |  |  |  |  |
|  |  |  |  |  |  | BS 6/9 |  |  |  |  |  |
|  |  |  |  |  |  | lymphoedema |  |  |  |  |  |
|  |  |  |  |  |  | atrophic scarring |  |  |  |  |  |
|  |  |  |  |  |  | iliacal tortuosity |  |  |  |  |  |
| **V3** | R745Q | M | Infrarenal | EVAR |  | pectus excavatum | FAA | yes | 1 normal (echo) | Father †71y rAAA, | not tested |
|  |  |  | Ascendens | TEVAR |  | BS 6/9 |  |  |  | multiple relatives CAA |  |
|  |  |  |  |  |  | coronairy tortuosity |  |  |  |  |  |
| **V4** | R745Q | F | Infrarenal | Open | rupture | varices, kyphosis | FAA | no | 3 normal (echo) | Paternal 3^rd^ degree 56y ascendens dilation | no |
|  |  |  |  | EVAR |  | wrist sign |  |  |  |  |  |
|  |  |  |  |  |  | BS 8/9 |  |  |  |  |  |
| **V5** | R745Q | M | Descendens | Open |  | skin hyperextensibility | FAA | yes | 6 normal (echo) | † Mother 80 y rAAA | not tested |
|  |  |  | Infrarenal | EVAR |  | translucent skin |  |  |  |  |  |
|  |  |  | Iliac |  |  |  |  |  |  |  |  |
|  |  |  | Popliteal (R) |  |  |  |  |  |  |  |  |
| **V12** | R745Q | M | Infrarenal | EVAR |  | varices | FAA | yes | 2 normal (echo) | † Father 77y rAAA | not tested |
|  |  |  | Iliac (L) | Open |  | inguinal hernia |  |  |  |  |  |
| **V13** | R745Q | M | Infrarenal | EVAR |  | velvety skin | FAA | yes | 1 normal (CT) | † Bother 65y Dissection | not tested |
|  |  |  | Iliac (L-R) |  |  | pectus excavatum |  |  | 3 normal (echo) | † Mother 82 AAA |  |
|  |  |  | Popliteal (L-R) |  |  | inguinal hernia, |  |  |  |  |  |
|  |  |  |  |  |  | span/length ratio 1.04 |  |  |  |  |  |
| **V14** | R745Q | M | Infrarenal | Open | Rupture 71y | inguinal hernia | SAA | yes | 1 nomal (echo) | none | not tested |
|  |  |  | Iliac (R) |  |  |  |  |  | 1normal (CT) |  |  |
| **V15** | R745Q | M | Infrarenal |  | Saccular | pectus excavatum | SAA | no | none | none | not tested |
| **V25** | R745Q | M | Infrarenal | FEVAR |  | Beighton 6 | SAA | yes | none | none | not tested |
|  |  |  | Ascendens |  |  |  |  |  |  |  |  |
|  |  |  | Arch |  |  |  |  |  |  |  |  |
|  |  |  | Iliac (L-R) |  |  |  |  |  |  |  |  |
| **V16** | R745Q | F | Infrarenal Ascendens | EVAR |  |  | FAA | yes | 4 normal (CT) | † Brother 76 y AAA, | not tested |
|  |  |  |  |  |  |  |  |  |  | Daughter with *TGFBR2* LPV fusiform aneurysm a. renalis |  |
| **V6** | V210A | M | Juxtarenal | Open | Rupture 43y | skin hyper- extensibility | FAA | yes | 1 Affected (CT) 2 normal CT | † Father 48y rAAA | not tested |
|  |  |  | Carotid int (R)(L) |  |  | inguinal hernia |  |  |  |  |  |
|  |  |  |  |  |  | severe arthrosis |  |  |  |  |  |
| **V7** | P169T | F | Ascendens |  |  | dystrophic scar | FAA | no | 4 normal (CT) | 1 Brother 64 y TAA | Brother |
|  |  |  |  |  |  | translucent skin |  |  |  | † Father AAA †Uncle AAA |  |
| **V8** | R468W | M | Infrarenal |  | Dissection 32 y | span/length ratio 1.06 | FAA | yes | none |  | not tested |
|  |  |  | Iliac (L)(R) |  |  |  |  |  |  |  |  |
| **V10** | D410N | M | Infrarenal | Open | Rupture 57y |  | FAA | no | 2 normal (echo) | † Father 77y AAA | not tested |
|  |  |  |  |  |  |  |  |  | 1 affected (CT) |  |  |
| **V11** | R693Q | M | Infrarenal | EVAR |  | scoliosis | FAA | no | 1 normal (echo) | † Mother 55y rAAA | no |
|  |  |  |  |  |  | span/length ratio 1.05 |  |  |  | Uncle EVAR 77y TAA/popllitea AA |  |
|  |  |  |  |  |  | varices |  |  |  |  |  |
| **V17** | R81C | M | Infrarenal |  |  |  | SAA | yes | none |  | not tested |
|  |  |  | Iliac (R) |  |  |  |  |  |  |  |  |
| **V9** | R81C | M | Infrarenal |  |  |  | FAA | yes | 2 normal (echo) | † Brother 55y rAAA | not tested |
|  |  |  | Iliac (L) |  |  |  |  |  |  |  |  |
|  |  |  |  |  |  |  |  |  |  |  |  |
| **V18** | T413I | F | Infrarenal | Open |  |  | SAA | yes | none |  | not tested |
|  |  |  | Arch | TEVAR |  |  |  |  |  |  |  |
| **V19** | R464W | M | Descendens | Open |  | scoliosis | FAA | yes | none | Brother 68 y AAA | Brother |
|  |  |  | Iliac (L) |  |  | inguinal hernia |  |  |  |  |  |
|  |  |  |  |  |  | muscle ruputure |  |  |  |  |  |
|  |  |  |  |  |  | varices |  |  |  |  |  |
| **V20** | E715Q | M | Infrarenal | EVAR |  |  | FAA | yes | 1 normal (CT) | † 2 Brothers AAA | not tested |
|  |  |  | Descendens | TEVAR |  |  |  |  |  | † Brother 86y AAA/ TAA |  |
|  |  |  | Femoral |  |  |  |  |  |  | Son 63y TAA |  |
| **V21** | D753N | M | Type B Dissection |  |  |  | SAA | yes | none |  | not tested |
|  |  |  | Root dilation |  |  |  |  |  |  |  |  |
| **V22** | A43V | F | Renal (L) |  | Saccular |  | SAA | yes | none |  | not tested |
|  |  |  | 3 Lienal |  |  |  |  |  |  |  |  |
| **V26** | A43V | F | Type B Dissection | TEVAR |  | translucent skin | FAA | no | none | Father 75y AAA | Father |
|  |  |  |  |  |  | jaw dislocation |  |  |  |  |  |
| **V27** | A43V | F | Arch saccular | Open | Arch elongation and tortuosity | skin translucency | SAA | yes | none |  | not tested |
|  |  |  | Vertebralis | Clipping |  | atrophic scaring |  |  |  |  |  |
|  |  |  | 2 Cerebral (SAB) |  |  | BS 6/9 |  |  |  |  |  |
| **V28** | A43V | M | Infrarenal | EVAR |  |  | SAA | no |  |  | not tested |
| **V23** | A139V | M | Infrarenal | EVAR |  | thin hyperelastic skin | SAA | yes | none |  | not tested |
|  |  |  | Iliac (R) |  |  |  |  |  |  |  |  |
| **V24** | A139V | M | Ascendens |  |  | varices | SAA | yes | 1 normal (CT) |  | not tested |
|  |  |  | Descendens |  |  | dystrophic scar |  |  |  |  |  |
| **V29** | T413I | M | Infrarenal | TEVAR | Rupture |  | FAA | yes | none | Brother 42 yr AAA | not tested |
|  |  |  | Ascendens |  |  |  |  |  |  |  |  |
|  |  |  |  |  |  |  |  |  |  |  |  |

Legend table S1: EVAR, endovascular aneurysm repair. TEVAR, Thoracic endovascular aneurysm repair. rAAA, ruptures AAA. TAA, thoracic aorta aneurysm. FAA, Familial aneurysm. SAA, sporadic aneurysm. CAA, cerebral aneurysm. BS, Beighton score. VUS, variant of unknown significance.# This patients had a likely pathogenic variant in LDLR c.131G>A, p.(Trp44*)
